# Gene expression in response to optical defocus of opposite signs reveals bidirectional mechanism of visually guided eye growth

**DOI:** 10.1371/journal.pbio.2006021

**Published:** 2018-10-09

**Authors:** Tatiana V. Tkatchenko, David Troilo, Alexandra Benavente-Perez, Andrei V. Tkatchenko

**Affiliations:** 1 Department of Ophthalmology, Columbia University, New York, New York, United States of America; 2 College of Optometry, State University of New York, New York, New York, United States of America; 3 Department of Pathology and Cell Biology, Columbia University, New York, New York, United States of America; Yale University, United States of America

## Abstract

Myopia (nearsightedness) is the most common eye disorder, which is rapidly becoming one of the leading causes of vision loss in several parts of the world because of a recent sharp increase in prevalence. Nearwork, which produces hyperopic optical defocus on the retina, has been implicated as one of the environmental risk factors causing myopia in humans. Experimental studies have shown that hyperopic defocus imposed by negative power lenses placed in front of the eye accelerates eye growth and causes myopia, whereas myopic defocus imposed by positive lenses slows eye growth and produces a compensatory hyperopic shift in refractive state. The balance between these two optical signals is thought to regulate refractive eye development; however, the ability of the retina to recognize the sign of optical defocus and the composition of molecular signaling pathways guiding emmetropization are the subjects of intense investigation and debate. We found that the retina can readily distinguish between imposed myopic and hyperopic defocus, and identified key signaling pathways underlying retinal response to the defocus of different signs. Comparison of retinal transcriptomes in common marmosets exposed to either myopic or hyperopic defocus for 10 days or 5 weeks revealed that the primate retina responds to defocus of different signs by activation or suppression of largely distinct pathways. We also found that 29 genes differentially expressed in the marmoset retina in response to imposed defocus are localized within human myopia quantitative trait loci (QTLs), suggesting functional overlap between genes differentially expressed in the marmoset retina upon exposure to optical defocus and genes causing myopia in humans. These findings identify retinal pathways involved in the development of myopia, as well as potential new strategies for its treatment.

## Author summary

The worldwide prevalence of myopia is predicted to increase from the current 23% to about 50% in the next three decades. Although much effort has been directed towards elucidating the mechanisms underlying refractive eye development and myopia, treatment options for myopia are mostly limited to optical correction, which does not prevent progression of myopia nor the pathological blinding complications often associated with the disease. Several experimental optics-based treatments have had only limited effect on myopia progression, and currently available drug treatments are limited and the mechanisms of action are not well understood. The development of safe and effective pharmacological treatments for myopia is urgently needed to prevent the impending myopia epidemic. The main obstacles that prevent the development of anti-myopia drugs are the uncertainties regarding the mechanisms controlling eye growth and optical development, including the molecular signaling pathways underlying it. In this study, we show that, contrary to the conventional thinking that myopic and hyperopic defocus trigger opposite changes in the same genes and pathways to guide postnatal eye growth, defocus of opposite signs affect eye growth via largely distinct retinal pathways. Knowing that myopic and hyperopic defocus signals drive eye growth in opposite directions and propagate via different pathways provides a framework for the development of new anti-myopia drugs. Myopia can be controlled pharmacologically by stimulating pathways underlying the retinal response to positive lenses and/or by suppressing pathways underlying the retinal response to negative lenses.

## Introduction

Myopia (nearsightedness) is an eye disorder characterized by blurred distance vision caused by negative refractive errors when the eye grows too long for its optical power. It is widespread [1], and the prevalence has been increasing around the world at an alarming rate, reaching 80%–90% in several parts of Asia [2–5]. Because of the increasing prevalence, myopia is rapidly becoming one of the leading causes of vision loss in several parts of the world, as the excessive eye growth associated with it often leads to serious vision-threatening complications, such as myopic maculopathy, chorioretinal atrophy, retinal tears, retinal detachment, myopic macular degeneration, and glaucoma [1,6–8]. It is estimated that 4.8 billion people (half of the world’s population) will be affected by myopia by 2050 [9], predicting an impending public health crisis. The World Health Organization designated myopia as one of five priority health conditions [10,11].

Developmental studies in humans and a wide variety of animals show that eyes grow into focus postnatally through a process called emmetropization. During emmetropization, the axial length of the eye grows to match its optical power, producing focused images on the retina; a mismatch between the optical power of the eye and its axial length leads to the development of hyperopia if eyes are too short for their optical power or myopia if eyes grow too long for their optical power. Visual experience is critical for this process, suggesting that postnatal eye growth is guided by visual feedback related to retinal defocus. Experimental studies in many animal species confirm the role of optical defocus in emmetropization, with observations that the eye can compensate for either imposed hyperopic or imposed myopic defocus by either increasing or decreasing its growth rate [12–26]. Myopia can be induced in species as diverse as primates, tree shrews, guinea pigs, mice, chickens, and fish by placing a negative lens in front of the eye and imposing hyperopic defocus on the retina [13,16,20,21,23,25–28]. Imposing myopic defocus on the retina with positive lenses, on the other hand, inhibits eye growth and produces a compensatory hyperopic shift in refraction in fish [28], chickens [21,23], and several mammalian species, including primates [13,16,18,20,25,26]. The balanced response to these two optical signals of opposite signs seems to be regulating eye growth and refractive development. Moreover, animal experiments in which the optic nerve was sectioned [29–31] and in which different parts of the retina were simultaneously exposed to the defocus of opposite signs [32–34] suggested that the process of emmetropization is largely controlled locally by the retina.

A number of studies have explored the cellular and molecular mechanisms of emmetropization, and while these studies have hinted at the involvement of various factors and pathways [35–44], the field has made only incremental gains and has been at a conceptual standstill for some time. Very little is actually known about the biological signaling that underlies the eye’s response to different signs of optical defocus, and the ability of the retina to detect the sign of defocus has been the subject of much controversy because of the lack of direct evidence for the underlying mechanism [19,45,46]. Essentially, two alternative models for the visual control of emmetropization have been proposed. In the first model, the rate of eye growth is regulated by the amount of retinal blur, regardless of the sign of defocus. In this model, more blur promotes more growth, and imposed hyperopic or myopic defocus could be compensated for appropriately because hyperopic eyes generally experience more defocus than myopic eyes, which experience some focus at near. If the retina indeed could not distinguish between myopic and hyperopic defocus, both imposed myopic and hyperopic defocus would be expected to affect expression of the same genes in the same direction. The second model suggests that postnatal eye growth is controlled by the sign of retinal defocus and that response to defocus is bidirectional, i.e., myopic defocus actively reduces growth and hyperopic defocus actively increases growth. This could be controlled by one controller that modulates growth rate or two separate controllers that work independently to increase or decrease growth. In the latter case, emmetropization is achieved through the balance between the two independent outputs. If this model is true, then imposed myopic and hyperopic defocus would be expected to modulate the expression of the same genes in opposite directions or affect expression of different sets of genes.

In this study, we analyzed whole-genome gene expression using massive parallel RNA sequencing (RNA-seq) in the retina of a New World primate, common marmosets (*Callithrix jacchus*), exposed to either myopic or hyperopic defocus. We demonstrate that primate retina (1) can distinguish between hyperopic and myopic optical defocus, and (2) responds to the different signs of defocus by activation or suppression of largely distinct pathways. These data support the hypothesis that emmetropization is regulated bidirectionally by two separate retinal controllers: one that involves active stimulation of eye growth by hyperopic defocus and another that actively suppresses eye growth in response to myopic defocus. We refer to this mechanism as Bidirectional Emmetropization by the Sign of Optical Defocus (BESOD). Furthermore, we identified key signaling pathways underlying the retinal responses to the defocus of different signs, which offer possible targets that can be manipulated pharmacologically to suppress myopia. These results establish the critical role of the retina in the control of postnatal eye growth and emmetropization, and provide a framework for the development of drugs that can be used to treat myopia and help prevent the blinding complications associated with it.

## Results

### Optical defocus induces large-scale changes in gene expression and affects numerous cellular functions and signaling pathways in the retina

**Fig 1:**
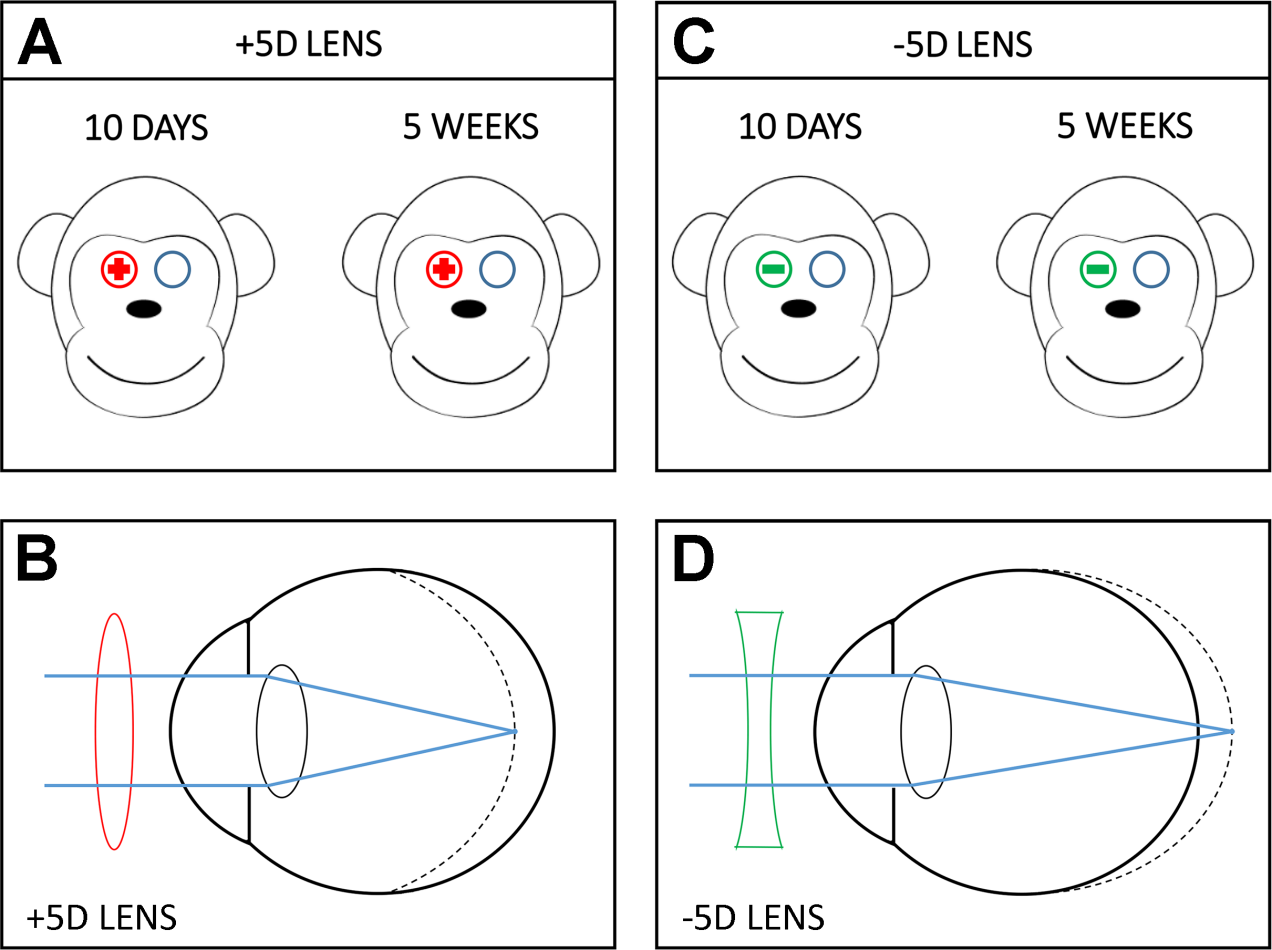
Experimental design and effect of positive and negative lenses on refractive eye development in marmosets. (A) Two groups of marmosets were treated with +5D lenses (red) for 10 days or 5 weeks; +5D lenses were applied to the right eyes, while contralateral left eyes served as controls and were fitted with plano lenses. (B) Positive lenses shift focal point in front of the retina and produce myopic defocus, which inhibits eye growth (dashed line). (C) Two groups of marmosets were treated with −5D lenses (green) for 10 days or 5 weeks; −5D lenses were applied to the right eyes, while contralateral left eyes served as controls and were fitted with plano lenses. (D) Negative lenses shift the focal point behind the retina and produce hyperopic defocus, which stimulates eye growth (dashed line).

The eyes of common marmosets compensate for optical defocus imposed by either negative or positive lenses by increasing or decreasing axial growth and developing myopic or hyperopic refractive errors, respectively [15,16,25,26]. To investigate the retina’s molecular responses to different signs of defocus, we analyzed changes in the whole-genome transcriptome in the retina of common marmosets (*C*. *jacchus*) exposed to myopic or hyperopic defocus using +5D or −5D single vision contact lenses, respectively. We applied the lenses to the right eye of 10–11-week-old (mean 74 ± 5 days) marmosets for 10 days to examine the effect of the initial exposure to retinal defocus, or for 5 weeks to explore the retinal responses when measurable changes in eye growth and refractive state typically just begin to be detected [25]. The left eye served as a control and was fitted with a plano contact lens (Fig 1). Table 1 provides descriptive optometric data of the subjects under each condition. The lens-imposed retinal defocus (the effective refractive error measured through the lens at the end of the rearing period) provides a measure of the average retinal defocus experienced by the subjects in each group at the time of the tissue collection. It shows that the +5D lenses imposed myopic defocus and the −5D lenses imposed hyperopic defocus after 10 days (early response) and 5 weeks (sustained response) of lens wear. As expected from earlier studies [25], there was little if any compensation for the imposed defocus observed for these treatment durations, as seen from the average interocular differences in spherical equivalent refractive error or vitreous chamber depth between the experimental and control eyes.

**Table 1:** Summary of biometric data for animals in the four experimental groups.

Treatment duration	Lens power (D)	Sample size (*N*)	Imposed defocus* (D)	Interocular difference*	
				RE (x-c, D)	VCD (x-c, mm)
10 days	+5D	3	−3.6 ± 1.6	+1.4 ± 1.6	−0.02 ± 0.06
10 days	−5D	3	+5.4 ± 0.8	+0.4 ± 0.8	−0.02 ± 0.04
5 weeks	+5D	3	−4.2 ± 1.4	+0.8 ± 1.4	−0.15 ± 0.06
5 weeks	−5D	3	+3.3 ± 1.9	−1.7 ± 1.9	+0.05 ± 0.07

*Measurements taken at the end of lens treatment.

Abbreviations: c, control eye; D, diopter; RE, refractive error; VCD, vitreous chamber depth; x, experimental eye.

**Fig 2:**
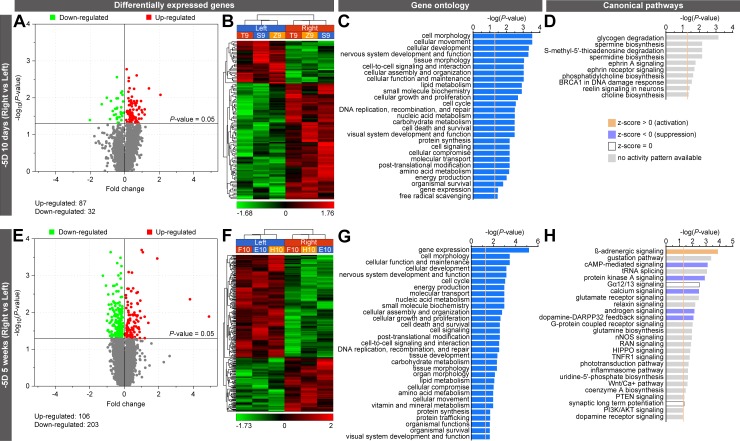
Hyperopic optical defocus induces large-scale changes in gene expression and signaling pathways in the retina. RNA-seq analysis of differential gene expression in two groups of marmosets exposed to −5D-lens-imposed hyperopic defocus for 10 days or 5 weeks. (A and E) Volcano plots showing differentially expressed genes. (B and F) Hierarchical clustering results showing that differentially expressed genes are organized into two clusters, i.e., genes that are down-regulated and genes that are up-regulated in the retina exposed to defocus. Right, −5D-lens-treated eye. Left, plano-lens-treated control eye. Letters and numbers above each column of the hierarchical clustering results indicate animal IDs. (C and G) Gene ontology analysis results showing biological processes affected by the differentially expressed genes. Vertical yellow line indicates *P* = 0.05. (D and H) Canonical signaling pathways affected by the differentially expressed genes. Vertical yellow line indicates *P* = 0.05. Z-score shows activation or suppression of the corresponding pathways. See S1, S2, S5, S6, S9, S10, S13, and S14 Tables for details. AKT, Serine and Threonine kinase AKT; BRCA1, breast cancer 1 early onset; cAMP, cyclic adenosine monophosphate; DARPP32, dopamine- and cAMP-regulated phosphoprotein 32 kDa; HIPPO, protein kinase Hippo; nNOS, neuronal nitric oxide synthase; PI3K, phosphatidylinositol 3-kinase; PTEN, phosphatase and tensin homolog; RAN, Ras-related nuclear protein; RNA-seq, massive parallel RNA sequencing; TNFR1, tumor necrosis factor receptor 1; tRNA, transfer RNA; Wnt, Wingless-integrated.

**Fig 3:**
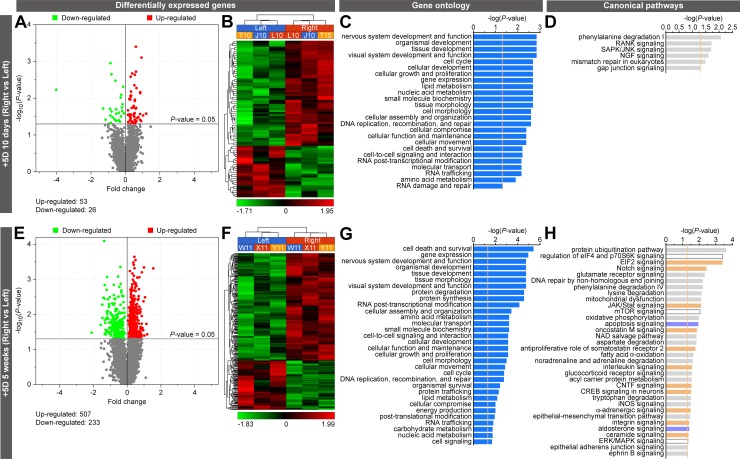
Myopic optical defocus induces large-scale changes in gene expression and signaling pathways in the retina. RNA-seq analysis of differential gene expression in two groups of marmosets exposed to +5D-lens-imposed myopic defocus for 10 days or 5 weeks. (A and E) Volcano plots showing differentially expressed genes. (B and F) Hierarchical clustering results showing that differentially expressed genes are organized into two clusters, i.e., genes that are down-regulated and genes that are up-regulated in the retina exposed to defocus. Right, +5D-lens-treated eye. Left, plano-lens-treated control eye. Letters and numbers above each column of the hierarchical clustering results indicate animal IDs. (C and G) Gene ontology analysis results showing biological processes affected by the differentially expressed genes. Vertical yellow line indicates *P* = 0.05. (D and H) Canonical signaling pathways affected by the differentially expressed genes. Vertical yellow line indicates *P* = 0.05. Z-score shows activation or suppression of the corresponding pathways. See S3, S4, S7, S8, S11, S12, S15, and S16 Tables for details. CNTF, ciliary neurotrophic factor; CREB, cAMP responsive element binding protein; EIF2, eukaryotic translation initiation factor 2; eIF4, eukaryotic initiation factor 4; ERK, extracellular signal-regulated kinase; iNOS, inducible nitric oxide synthase; JAK, Janus kinase; JNK, c-Jun N-terminal kinase; MAPK, mitogen-activated protein kinase; mTOR, mammalian target of rapamycin; NAD, nicotinamide adenine dinucleotide; NGF, nerve growth factor; p70S6K, ribosomal protein S6 kinase; RANK, receptor activator of nuclear factor kappa-B; RNA-seq, massive parallel RNA sequencing; SAPK, stress-activated protein kinase; Stat, signal transducer and activator of transcription protein.

Following lens treatment, retinae were collected and used to perform whole-genome gene expression profiling using RNA-seq. In the eyes treated with −5D lenses for 10 days, a total of 119 genes, organized into 6 genetic networks, were differentially expressed, compared with controls (Fig 2A and 2B; S1 and S5 Tables; S1 Fig). Specifically, 87 genes were up-regulated and 32 genes were down-regulated. After 5 weeks of exposure to −5D lenses, 309 genes were differentially expressed compared to controls, and these genes were organized into 17 genetic networks (Fig 2E and 2F; S2 and S6 Tables; S2–S4 Figs): 106 genes were up-regulated and 203 genes were down-regulated. In the +5D-lens-treated animals, 79 genes, organized into 4 genetic networks, were differentially expressed after 10 days (Fig 3A and 3B; S3 and S7 Tables; S5 Fig), and 740 genes, organized into 25 genetic networks, were differentially expressed after 5 weeks of treatment (Fig 3E and 3F; S4 and S8 Tables; S6–S10 Figs), compared with the control eyes. In the animals treated with +5D lenses, a total of 53 genes were up-regulated and 26 genes were down-regulated after 10 days, whereas 507 genes were up-regulated and 233 genes were down-regulated after 5 weeks of treatment.

In terms of the numbers of differentially expressed genes, negative-lens-imposed hyperopic defocus induced a stronger response at the level of gene expression compared to the positive-lens-imposed myopic defocus during the first 10 days of treatment, while positive lenses elicited a substantially stronger response compared to negative lenses after 5 weeks of treatment.

Gene ontology (GO) analysis revealed that optical defocus affects numerous cellular functions, with noticeable differences between marmoset eyes exposed to negative and positive lenses, as well as between treatment durations of 10 days and 5 weeks (Fig 2C and 2G; S9 and S10 Tables; Fig 3C and 3G; S11 and S12 Tables). This initial observation was further reinforced by the analysis of canonical pathways affected by the positive and negative lenses (Fig 2D and 2H; S13 and S14 Tables; Fig 3D and 3H; S15 and S16 Tables). We found that the early response (10 days) to hyperopic defocus imposed by −5D lenses primarily involved pathways regulating glycogen degradation, ephrin and reelin signaling, as well as biosynthesis of spermine and choline (Fig 2D; S13 Table), while the sustained response (5 weeks) involved activation of ß-adrenergic signaling and suppression of cAMP-mediated signaling, protein kinase A signaling, calcium signaling, androgen signaling, and dopamine-DARPP32 feedback signaling, among several other pathways (Fig 2H; S14 Table). On the other hand, the early response to myopic defocus imposed by +5D lenses primarily involved phenylalanine degradation and RANK, SAPK/JNK, NGF, and gap junction signaling (Fig 3D; S15 Table), while the sustained response involved activation of EIF2, Notch, JAK/Stat, oncostatin M, somatostatin receptor 2, interleukin, CNTF, CREB, α-adrenergic, integrin, and ceramide signaling as well as suppression of apoptosis and aldosterone signaling (Fig 3H; S16 Table). Collectively, these results demonstrate that optical defocus causes large-scale changes in gene expression controlling metabolism and cell signaling in the retina, and that there are substantial differences in the retinal response to hyperopic and myopic defocus.

### There is very little overlap between genes differentially expressed in response to hyperopic or myopic defocus after 10 days or 5 weeks

**Fig 4:**
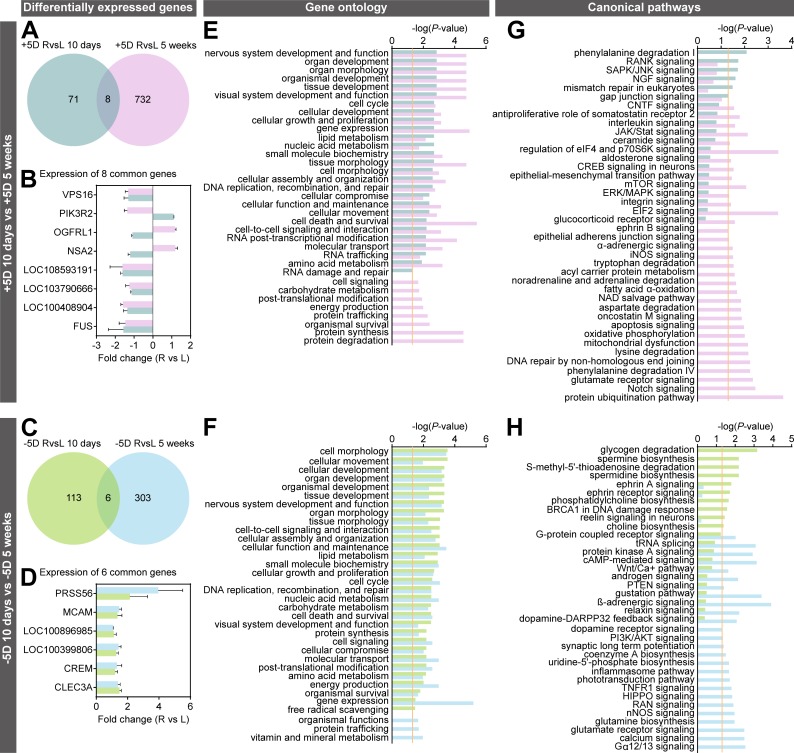
Optical defocus affects different genes and signaling pathways in the retina after 10 days and 5 weeks of exposure. Comparison of differential genes and pathways affected by imposed hyperopic or myopic defocus after 10 days and 5 weeks of exposure. (A and C) Venn diagrams showing very little overlap between genes differentially expressed after 10 days or 5 weeks of exposure to defocus. (B and D) Graphs showing expression of genes differentially expressed both after 10 days and after 5 weeks of exposure to defocus. Error bars, SD. R, +5D- or −5D-lens-treated eye. L, plano-lens-treated control eye. (E and F) Comparison of biological processes affected by either positive or negative defocus after 10 days or 5 weeks of exposure. Vertical yellow line indicates *P* = 0.05. (G and H) Comparison of canonical signaling pathways affected by either positive or negative defocus after 10 days or 5 weeks of exposure. Vertical yellow line indicates *P* = 0.05. Colors indicate experimental marmoset groups and correspond to the colors in Venn diagrams. See S9–S18 Tables for details. AKT, Serine and Threonine kinase AKT; BRCA1, breast cancer 1 early onset; cAMP, cyclic adenosine monophosphate; CNTF, ciliary neurotrophic factor; CREB, cAMP responsive element binding protein; DARPP32, dopamine- and cAMP-regulated phosphoprotein 32 kDa; EIF2, eukaryotic translation initiation factor 2; eIF4, eukaryotic initiation factor 4; ERK, extracellular signal-regulated kinase; HIPPO, protein kinase Hippo; iNOS, inducible nitric oxide synthase; JAK, Janus kinase; JNK, c-Jun N-terminal kinase; L, left; MAPK, mitogen-activated protein kinase; mTOR, mammalian target of rapamycin; NAD, nicotinamide adenine dinucleotide; NGF, nerve growth factor; nNOS, neuronal nitric oxide synthase; PI3K, phosphatidylinositol 3-kinase; PTEN, phosphatase and tensin homolog; p70S6K, ribosomal protein S6 kinase; R, right; RAN, Ras-related nuclear protein; RANK, receptor activator of nuclear factor kappa-B; SAPK, stress-activated protein kinase; Stat, signal transducer and activator of transcription protein; TNFR1, tumor necrosis factor receptor 1; tRNA, transfer RNA; Wnt, Wingless-integrated.

**Fig 5:**
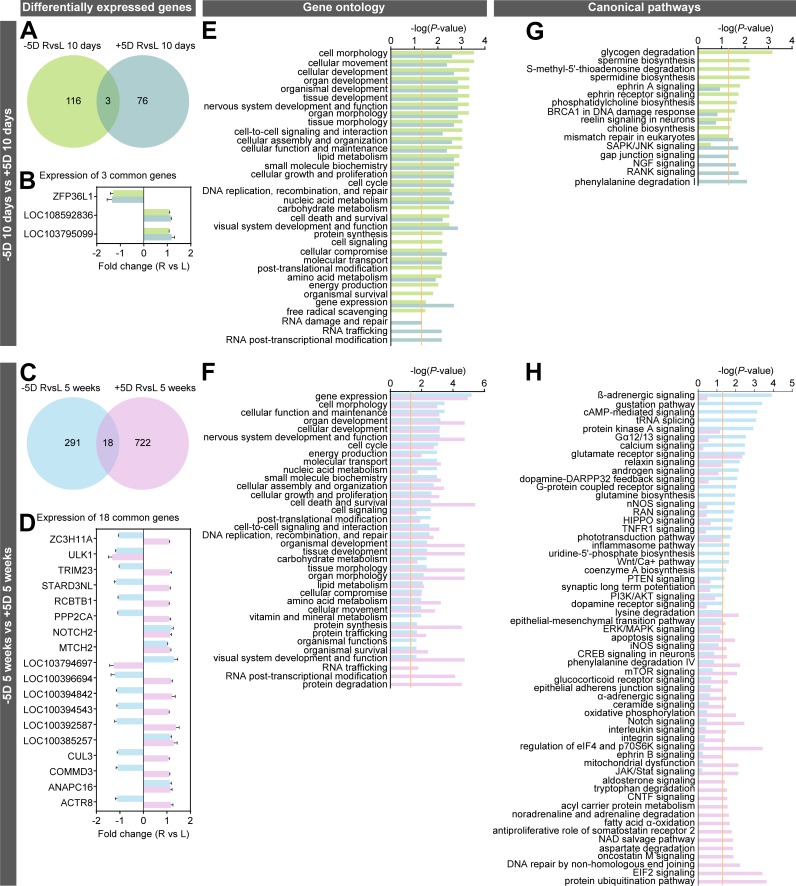
Myopic and hyperopic defocus affect different genes and signaling pathways in the retina. Comparison of differential genes and signaling pathways affected by either +5D-lens-imposed myopic defocus or −5D-lens-imposed hyperopic defocus. (A and C) Venn diagrams showing very little overlap between genes differentially expressed after exposure to −5D or +5D lenses. (B and D) Graphs showing expression of genes differentially expressed both after exposure to −5D and +5D lenses. Error bars, SD. R, −5D- or +5D-lens-treated eye. L, plano-lens-treated control eye. (E and F) Comparison of biological processes affected by either −5D or +5D lenses. Vertical yellow line indicates *P* = 0.05. (G and H) Comparison of canonical signaling pathways affected by either −5D or +5D lenses. Vertical yellow line indicates *P* = 0.05. Colors indicate experimental marmoset groups and correspond to the colors in Venn diagrams. See S9–S16, S19, and S20 Tables for details. AKT, Serine and Threonine kinase AKT; BRCA1, breast cancer 1 early onset; cAMP, cyclic adenosine monophosphate; CNTF, ciliary neurotrophic factor; CREB, cAMP responsive element binding protein; DARPP32, dopamine- and cAMP-regulated phosphoprotein 32 kDa; EIF2, eukaryotic translation initiation factor 2; eIF4, eukaryotic initiation factor 4; ERK, extracellular signal-regulated kinase; HIPPO, protein kinase Hippo; iNOS, inducible nitric oxide synthase; JAK, Janus kinase; JNK, c-Jun N-terminal kinase; L, left; MAPK, mitogen-activated protein kinase; mTOR, mammalian target of rapamycin; NAD, nicotinamide adenine dinucleotide; NGF, nerve growth factor; nNOS, neuronal nitric oxide synthase; PI3K, phosphatidylinositol 3-kinase; PTEN, phosphatase and tensin homolog; p70S6K, ribosomal protein S6 kinase; R, right; RAN, Ras-related nuclear protein; RANK, receptor activator of nuclear factor kappa-B; SAPK, stress-activated protein kinase; Stat, signal transducer and activator of transcription protein; TNFR1, tumor necrosis factor receptor 1; tRNA, transfer RNA; Wnt, Wingless-integrated.

Taking into account the differences in GO functions and signaling pathways involved in the defocus response in the four experimental groups, we additionally performed a systematic comparison of cellular functions and canonical pathways affected in the different experimental groups (Figs 4 and 5; S9–S20 Tables). This analysis revealed that each experimental condition leads to differential expression of a unique set of genes, with very little overlap between the datasets (Fig 4A and 4C; Fig 5A and 5C). There was only an eight-gene overlap between the +5D/10-days group and the +5D/5-weeks group, with three genes (*PIK3R2*, *OGFRL1*, and *NSA2*) exhibiting differential expression in opposite directions (Fig 4B; S17 Table). Six genes were common between the −5D/10-days and −5D/5-weeks groups, with all genes expressed in the same direction in both groups (Fig 4D; S18 Table). Three genes were common between the −5D/10-days and +5D/10-days groups, all expressed in the same direction (Fig 5B; S19 Table), indicating that they were sensitive to defocus but not to the sign of defocus. However, 13 out of 18 genes, which were common between the −5D/5-weeks and +5D/5-weeks groups, exhibited sign-of-defocus–specific expression (Fig 5D; S20 Table). These included nine coding genes (*ZC3H11A*, *TRIM23*, *STARD3NL*, *RCBTB1*, *PPP2CA*, *LOC100394842*, *CUL3*, *COMMD3*, *ACTR8*) and four long noncoding RNAs (*LOC103794697*, *LOC100396694*, *LOC100394543*, *LOC100392587*). Thus, these data reveal that hyperopic and myopic defocus affect the expression of largely different genes in the retina. Very few genes are affected by both hyperopic and myopic defocus and change direction of expression in response to the defocus of different sign.

### Early and sustained retinal responses to defocus are guided by largely distinct pathways

GO analysis showed that the early (10 days) and sustained (5 weeks) response to defocus involved largely the same cellular functions (albeit to a different extent) (Fig 4E and 4F; S9–S12 Tables); however, there were several cellular functions unique to the sustained response to defocus (Fig 4E and 4F; S9–S12 Tables). Specifically, the response to 5 weeks of myopic defocus imposed by +5D lenses (associated with reduced axial eye growth) involved changes in metabolism, cell signaling, protein trafficking, and posttranslational modification of proteins (Fig 4E; S11 and S12 Tables), whereas the sustained response to hyperopic defocus imposed by −5D lenses (associated with increased axial eye growth) involved substantial increase in gene expression, protein trafficking, and vitamin and mineral metabolism (Fig 4F; S9 and S10 Tables). Moreover, pathway analysis revealed almost complete transition from one set of pathways to another between 10 days of treatment and 5 weeks of treatment for both −5D and +5D experimental groups (Fig 4G and 4H; S13–S16 Tables). For myopic defocus imposed by positive lenses, the most prominent change is a transition from RANK, SAPK/JNK, NGF, and gap junction signaling to protein ubiquitination pathway, Notch signaling, glutamate receptor signaling, oncostatin M signaling, α-adrenergic signaling, and ephrin B signaling (Fig 4G; S15 and S16 Tables). For hyperopic defocus imposed by negative lenses, there is an apparent transition from ephrin A and reelin signaling to ß-adrenergic signaling, Gα12/13 signaling, calcium signaling, glutamate receptor signaling, TNFR1 signaling, HIPPO signaling, RAN signaling, NOS signaling, synaptic long-term potentiation, relaxin signaling, and dopamine receptor signaling (Fig 4H; S13 and S14 Tables). In summary, there is almost complete transition from one set of pathways to another over time when the retina responds to sustained optical defocus.

### Different pathways guide retinal responses to hyperopic and myopic defocus associated with changes in eye growth

Comparison of the cellular functions affected by different defocus in the −5D and +5D experimental groups revealed that although most cellular functions affected by imposed hyperopic or myopic defocus were the same (Fig 5E and 5F; S9–S12 Tables), there was a clear increase in gene expression during the first 10 days after exposure to myopic defocus imposed by +5D lenses and a substantial increase in RNA trafficking and RNA posttranscriptional modifications after exposure to +5D lenses in both 10-days and 5-weeks groups (Fig 5E and 5F; S9–S12 Tables), indicating that alternative splicing and isoform switching play important roles in the eye’s response to positive lenses. Furthermore, pathway analysis revealed that positive and negative lenses influenced different sets of pathways (Fig 5G and 5H; S13–S16 Tables).

During the first 10 days, the retina responded to hyperopic defocus imposed by −5D lenses with changes in glycogen and S-methyl-5′-thioadenosine degradation, spermine and choline biosynthesis, and ephrin receptor signaling, whereas myopic defocus imposed by +5D lenses caused changes in phenylalanine degradation, RANK, NGF, and gap junction signaling (Fig 5G; S13 and S15 Tables).

Five weeks of exposure to optical defocus resulted in large-scale changes in retinal signaling. The −5D-lens-imposed hyperopic defocus primarily affected ß-adrenergic and cAMP-mediated signaling, tRNA splicing, protein kinase A signaling, Gα12/13 signaling, calcium signaling, dopamine-DARPP32 feedback signaling, G-protein coupled receptor signaling, glutamine biosynthesis, nNOS signaling, RAN, HIPPO, and TNFR1 signaling as well as the Wnt/Ca+ pathway, among others (Fig 5H; S14 and S16 Tables). Retinal response to 5 weeks of myopic defocus imposed by +5D lenses involved protein translation and protein ubiquitination pathways, oncostatin M and somatostatin receptor 2 signaling, CNTF signaling, aldosterone signaling, JAK/Stat signaling, ephrin B signaling, integrin signaling, interleukin signaling, Notch signaling, α-adrenergic signaling, glucocorticoid receptor signaling, and mTOR signaling, among several other pathways (Fig 5H; S14 and S16 Tables).

Taken together, these data suggest that postnatal eye growth and refractive development are regulated by a bidirectional mechanism that involves active stimulation of eye growth by hyperopic defocus and active suppression of eye growth by myopic defocus, through largely independent pathways.

### Many genes differentially expressed in response to optical defocus in marmosets localize within human myopia QTLs

**Fig 6:**
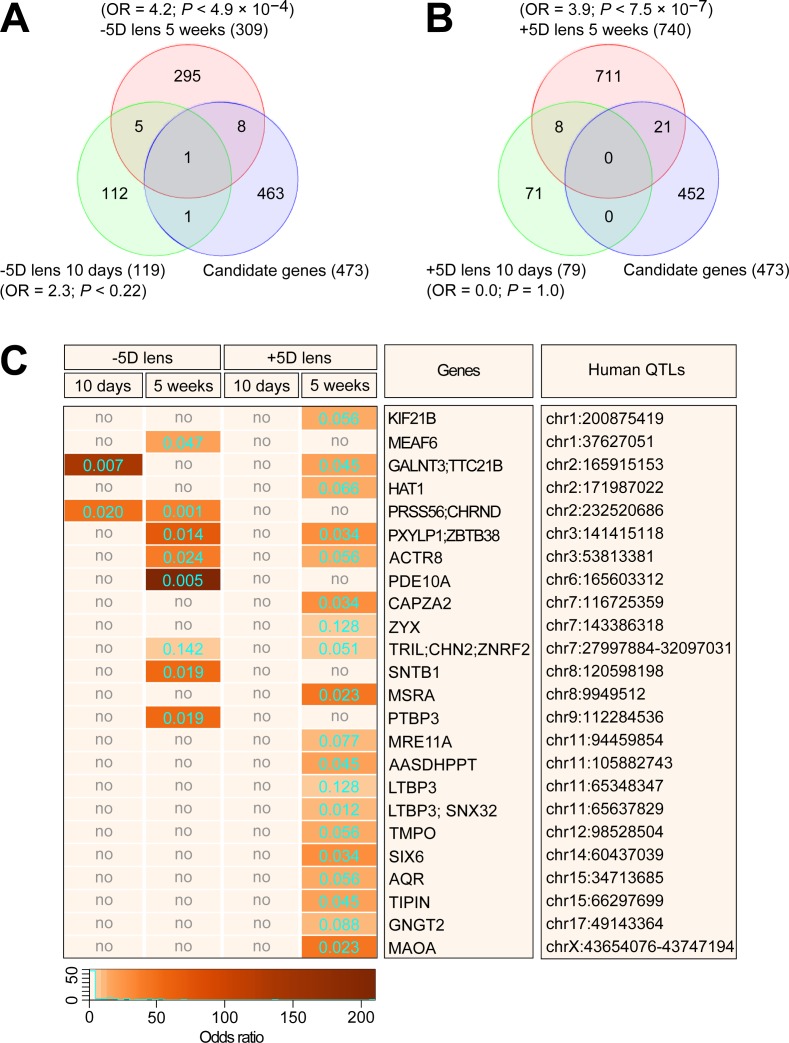
Genes differentially expressed in the retina of marmosets exposed to optical defocus are localized within human QTLs associated with myopia. (A) Venn diagram showing overlap between genes localized within human myopia QTLs and genes differentially expressed in the retina of marmosets exposed to hyperopic defocus imposed by −5D lenses. (B) Venn diagram showing overlap between genes localized within human myopia QTLs and genes differentially expressed in the retina of marmosets exposed to myopic defocus imposed by +5D lenses. (C) Heatmap depicting genes, odds ratios, and corresponding *P* values for the overlaps between specific QTLs and genes differentially expressed in the retina of marmosets exposed to optical defocus. Colors indicate odds ratios and numbers within each cell indicate *P* values. See S21–S24 Tables for details. no, no overlap; OR, odds ratio; QTL, quantitative trait locus.

Genes comprising signaling pathways that underlie physiological processes are often targeted by mutations, causing human diseases. To identify candidate genes involved in myopia development in humans, we compared the genes differentially expressed in the marmoset retina in response to imposed defocus with a list of genes located within QTLs found to be associated with myopia in the human population (S21 Table) [47]. This analysis revealed that a total of 29 differential genes identified in this study were localized within 24 human myopia QTLs (Fig 6; S22–S24 Tables), including two genes differentially expressed in the −5D/10-days group (Fig 6A and 6C), nine genes differentially expressed in the −5D/5-weeks group (Fig 6A and 6C), and 21 genes differentially expressed in the +5D/5-weeks group (Fig 6B and 6C). The overlap between myopia candidate genes and genes differentially expressed in the −5D/5-weeks and +5D/5-weeks groups was statistically significant (OR = 4.2, *P* < 4.9 × 10^−4^; OR = 3.9, *P* < 7.5 × 10^−7^, respectively), indicating functional connection between genes found by the genetic mapping studies in humans and genes differentially expressed in the marmoset retina upon exposure to optical defocus. These data also suggest that approximately 24% of the human QTLs may be associated with genetic variations in the genes identified in marmosets as regulating retinal response to defocus.

## Discussion

While there is plentiful evidence that eye growth can be modulated by imposed optical defocus of different signs, there remains little consensus regarding the mechanism underlying the eye’s sensitivity to the sign of optical defocus. Nevin and colleagues [24] reported that preventing access to sharp vision prevented refractive compensation for the sign of defocus, arguing that eye growth responds to blur and not the sign of defocus. Conversely, other findings support the alternative view that the eye can detect the sign of defocus and adjust its growth accordingly to compensate for either hyperopic or myopic defocus even when imposed on an image that is significantly blurred, indirectly supporting the BESOD model of emmetropization [48–50]. Furthermore, the sign-of-defocus–sensitive expression of the retinal transcription factor *ZENK/Egr1* led to the hypothesis that both myopic and hyperopic defocus would affect expression of the same genes in the retina but in opposite direction, presumably activating or suppressing a single pathway controlling eye growth [51].

**Fig 7:**
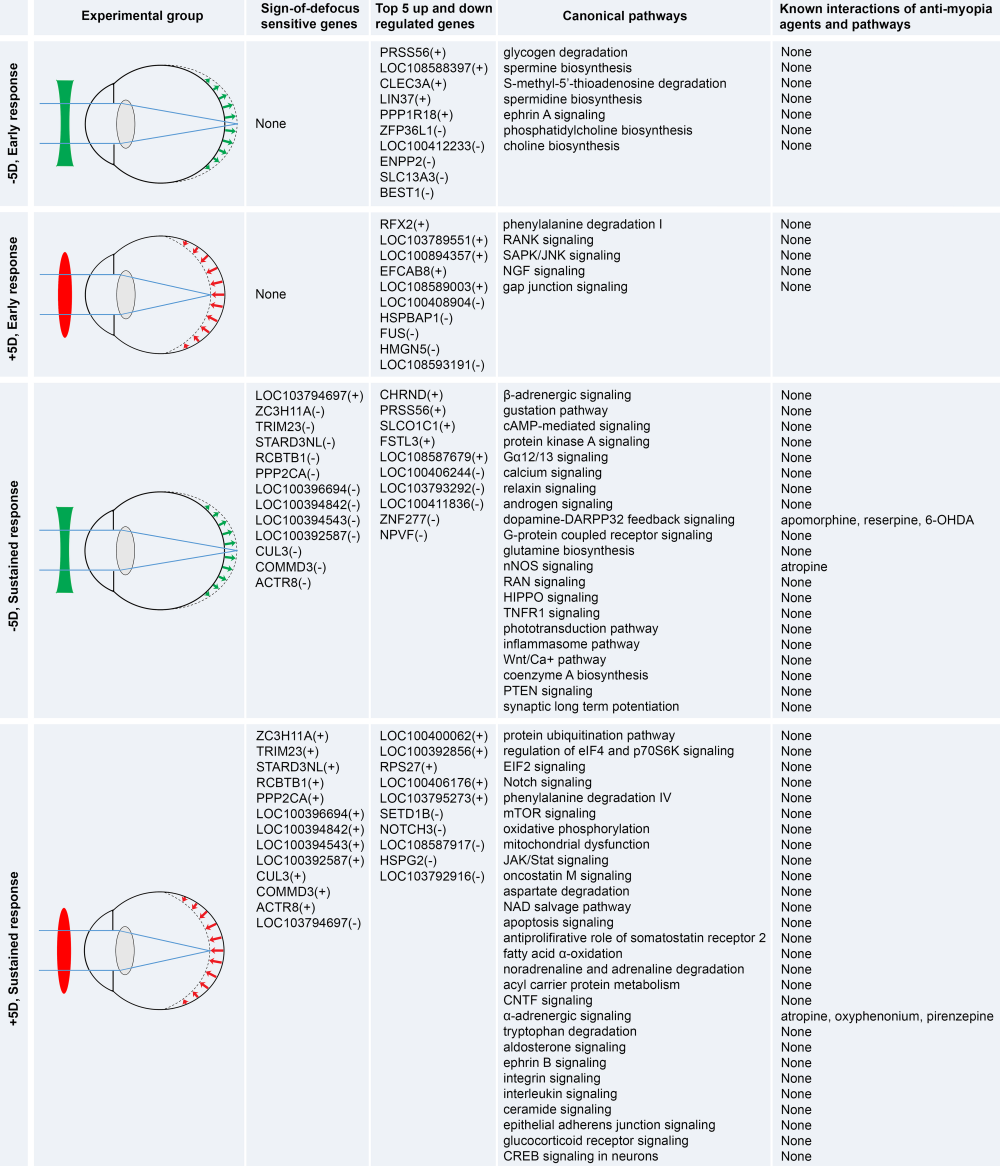
Summary of the key genes and pathways underlying BESOD. Our findings support the view that emmetropization is regulated by a bidirectional growth control mechanism, which involves active stimulation of eye growth by hyperopic defocus and active suppression of eye growth by myopic defocus (first column). We find that these two signals propagate in the retina via largely distinct (possibly even independent) pathways. Early response to optical defocus involves different sets of genes, with no genes exhibiting sign-of-defocus–sensitive expression. Although the sustained response also involves largely different sets of genes, several genes (listed in the second column) exhibit sign-of-defocus–sensitive expression. We speculate that they may act as switches that trigger largely distinct sign-of-defocus–specific signaling cascades underlying response to the defocus of opposite signs. We identified a total of 819 genes differentially expressed in the retina exposed to myopic defocus and 428 genes differentially expressed in the retina exposed to hyperopic defocus. The top five up- and down-regulated genes in each condition are shown in the third column. Some of over 100 canonical pathways involved in the regulation of the retinal response to defocus of opposite signs are listed in the fourth column. Three pathways have been previously targeted pharmacologically for myopia control (fifth column): (1) dopamine signaling was suggested to be a target for apomorphine, reserpine, and 6-OHDA [52]; (2) nitric oxide signaling was implicated in the anti-myopic atropine effect [53]; and (3) α-adrenergic signaling was suggested to be a target of atropine, oxyphenonium, and pirenzepine [54]. BESOD, Bidirectional Emmetropization by the Sign of Optical Defocus; cAMP, cyclic adenosine monophosphate; CREB, cAMP responsive element binding protein; CNTF, ciliary neurotrophic factor; DARPP32, dopamine- and cAMP-regulated phosphoprotein 32 kDa; EIF2, eukaryotic translation initiation factor 2; eIF4, eukaryotic initiation factor 4; HIPPO, protein kinase Hippo; JAK, Janus kinase; JNK, c-Jun N-terminal kinase; mTOR, mammalian target of rapamycin; NAD, nicotinamide adenine dinucleotide; NGF, nerve growth factor; nNOS, neuronal nitric oxide synthase; PTEN, phosphatase and tensin homolog; p70S6K, ribosomal protein S6 kinase; RAN, Ras-related nuclear protein; RANK, receptor activator of nuclear factor kappa-B; SAPK, stress-activated protein kinase; Stat, signal transducer and activator of transcription protein; TNFR1, tumor necrosis factor receptor 1; Wnt, Wingless-integrated; 6-OHDA, 6-hydroxydopamine.

Our data provide direct evidence that the retina detects myopic and hyperopic defocus separately through largely distinct and independent pathways, supporting the existence of BESOD (Fig 7). Furthermore, we found that there is very little overlap between the genes and pathways underlying response to the myopic defocus and the genes and pathways underlying response to the hyperopic defocus. We found only 13 genes that exhibited sign-of-defocus–sensitive expression, i.e., *ACTR8*, *COMMD3*, *CUL3*, *LOC100392587*, *LOC100394543*, *LOC100394842*, *LOC100396694*, *LOC103794697*, *PPP2CA*, *RCBTB1*, *STARD3NL*, *TRIM23*, and *ZC3H11A*. These genes represent about 1% of all genes affected by the optical defocus in the retina, and we speculate that they may act as switches that trigger largely distinct sign-of-defocus–specific signaling cascades underlying BESOD.

Our findings also support the existence of a switch from one set of genes to another when the retina transitions from an early response to defocus to a later, possibly sustained, response. There was almost complete transition from one set of pathways at 10 days to different pathways after 5 weeks of treatment with both myopic and hyperopic defocus imposed by the positive and negative lenses, respectively. The transition from the early response to the later response in the retinae exposed to myopic defocus was associated with significant changes in cell signaling, protein synthesis and degradation, posttranslational modification of proteins, protein trafficking, and energy production. This was accompanied by the transition from the pathways regulating phenylalanine degradation, RANK, SAPK/JNK, NGF, and gap junction signaling to the pathways that regulate ephrin B, α-adrenergic, iNOS, fatty acid α-oxidation, oncostatin M, glutamate receptor, Notch, and protein ubiquitination signaling, among other pathways. In the retinae exposed to hyperopic defocus, transition from the early response to the later response was associated with changes in gene expression, protein trafficking, and vitamin and mineral metabolism. It was accompanied by the transition from the pathways regulating glycogen degradation, spermine and spermidine biosynthesis, ephrin A and reelin signaling to the pathways that control synaptic long-term potentiation and phototransduction as well as dopamine receptor, TNFR1, HIPPO, RAN, nNOS, glutamate receptor, calcium, and ß-adrenergic signaling, among other pathways. We also found that although response to negative lenses appeared to be stronger compared to positive lenses during the first 10 days of exposure, positive lenses elicited a substantially stronger response at the level of gene expression after 5 weeks of treatment. This suggests that the time course of retinal response to hyperopic and myopic defocus might be slightly different and that positive lenses ultimately may have greater impact on eye growth than negative lenses.

Our data also suggest that positive and negative lenses affect different sets of genes and pathways. The most significant differences between positive and negative lenses after 10 days of treatment were related to the cellular functions underlying RNA trafficking and RNA splicing, which may indicate that initial exposure to defocus activates a large-scale switch from one set of protein isoforms to another. Moreover, negative lenses primarily affected glycogen degradation, spermine and spermidine biosynthesis, and ephrin A and reelin signaling, whereas positive lenses induced changes in gap junction, NGF, and RANK signaling as well as phenylalanine degradation. Positive and negative lenses also affected significantly different cellular functions and pathways after 5 weeks of treatment. The largest difference between the 5-weeks groups were significant changes in RNA trafficking and splicing induced by positive lenses, which again suggests a large-scale switch from one set of protein isoforms to another. While negative lenses primarily affected pathways regulating ß-adrenergic signaling, cAMP-mediated signaling, calcium signaling, dopamine signaling, G-protein coupled receptor signaling, glutamine biosynthesis, nNOS signaling, RAN signaling, HIPPO signaling, TNFR1 signaling, Wnt/Ca+ pathway, and synaptic long-term potentiation, positive lenses caused changes in pathways regulating protein ubiquitination, fatty acid α-oxidation, and tryptophan degradation as well as EIF2, oncostatin M, CNTF, aldosterone, JAK/Stat, ephrin B, integrin, Notch, glucocorticoid receptor, mTOR, iNOS, and α-adrenergic signaling, among other pathways.

Although many pathways that we found to underlie the retinal responses to defocus are novel, there are several pathways that had previously been implicated in the development of myopia. For example, a mutation in the gene *NDUFAF7*, which encodes a mitochondrial reduced nicotinamide adenine dinucleotide (NADH) dehydrogenase complex assembly factor, was associated with pathological myopia in humans, implicating mitochondrial dysfunction in refractive error development [55]. Protein ubiquitination may be associated with myopia development, because a loss-of-function mutation in the E3 ubiquitin-protein ligase *UBE3B* was shown to cause Kaufman oculocerebrofacial syndrome, which includes myopia as one its prominent features [56]. Two genome-wide association studies found a strong association between refractive error and *GJD2*, which encodes gap junction protein 2 expressed in the retina, suggesting that gap junction signaling may be involved in refractive eye development [57,58]. Glutamate receptor signaling was found to be involved in myopia development by two genetic linkage studies [58,59] and also in a study of a glutamate receptor *Grik2* mouse knockout model [36]. Aspartate receptors were implicated in myopia development by at least two studies [60,61]. Nitric oxide and cAMP signaling were also suggested to play a role in the development of myopia by several studies in chickens [37–39]. Dopamine signaling has long been associated with experimental myopia and refractive eye development [40]. Riddell and colleagues also found that pathways regulating apoptosis, oxidative phosphorylation, and mesenchymal development are involved in refractive development in chickens and humans [41,43]. Our finding that myopic and hyperopic defocus signals driving eye growth in opposite directions propagated via largely different retinal pathways is supported by several functional studies, which found that pharmacological agents that inhibit negative-lens-induced myopia are completely different from those inhibiting positive-lens-induced hyperopia [62–64]. Moreover, the temporal features of flickering light that suppress refractive error development are different for imposed myopia and hyperopia [65]. We also note that many of the genes changing expression in response to retinal defocus in this study are localized within human QTLs linked to myopia. This strongly suggests that there is a functional overlap between the genes that regulate retinal response to defocus and the genes that cause human myopia.

What creates conditions for the development of myopia and why myopia progresses in some individuals and not in others remain important topics of further research. Several treatment options for myopia, such as optical correction using spectacles, multifocal contact lenses, or atropine, are available, but efficacy is limited [66–72]. The identification of drug targets and the development of suitable drugs that can be used to suppress or possibly even reverse the progression of myopia require a deeper understanding of the signaling pathways underlying the ocular response to optical defocus of different signs. The results of this study show that the retina can distinguish between myopic and hyperopic defocus and responds to defocus of opposite signs by activating largely distinct pathways. Identification of these pathways provides a framework for understanding the molecular mechanisms underlying the visual control of emmetropization and the identification of new drug targets for the development of more effective treatment options for myopia.

## Materials and methods

### Ethics statement

Marmosets were bred and raised in the animal care facilities at the New England College of Optometry and at the State University of New York, College of Optometry, according to the United States Department of Agriculture (USDA) standards for animal care and use, and fully complied with the guidelines outlined in the Weatherall report on the use of nonhuman primates in research. All procedures also adhered to the Association for Research in Vision and Ophthalmology (ARVO) statement for the use of animals in ophthalmic and vision research and were approved by the New England College of Optometry and SUNY College of Optometry Institutional Animal Care and Use Committees (Protocol Nos. DT-1.10.07 and DT-2011-06-1, respectively). Animals were anesthetized via intramuscular injection of alfaxalone (1.5 mg/kg) and were humanely killed by intracardiac pentobarbital (100 mg/kg) overdose while under deep surgical anesthesia.

### Animals and lens treatment

Twelve common marmosets (*C*. *jacchus*) were used in this study. Marmosets were housed under lighting provided from daylight balanced fluorescent lamps on a 10-hour:14-hour light-dark cycle. Temperature was maintained at 75 ± 2°F with 45% ± 5% humidity. Food and water were provided ad libitum within the home cage and consisted of a formulated dry pellet (Mazuri New World Diet, Mazuri Exotic Animal Nutrition, St. Louis, MO) with regularly varied supplements of fresh fruit and protein. Custom-made soft single vision contact lenses were used to impose defocus. Animals of both sexes, which were 74 ± 5 days old at the beginning of the experiment, were fitted with either a +5D (imposed myopic defocus) or −5D (imposed hyperopic defocus) contact lens on one eye for either 10 days or 5 weeks. The contralateral eye wore a plano lens and was used as an interocular control. All lenses were inserted at the beginning of the light period and removed daily at the beginning of the dark period. Changes in axial eye growth and refractive state were measured at the onset and at the end of lens treatment. Refractive state was measured by retinoscopy and Hartinger coincidence refractometry following cycloplegia with 1% cyclopentolate. Refractive state was taken as the average of the spherical equivalents from both measures. Axial length was measured with A-scan ultrasonography and reported as changes in vitreous chamber depth (on-axis distance from the posterior surface of the crystalline lens to the inner surface of the retina). Our goal was to explore gene expression during detection of and active compensation for imposed defocus, when biometric changes cannot be readily detected. Our selection of the 10-day and 5-week rearing durations was guided by our previously reported data from two independent groups of age-matched marmosets (eight animals each), which were raised exposed to either imposed myopic defocus (with +5D lenses) or imposed hyperopic defocus (with −5D lenses) until they achieved full compensation. In these animals, we obtained the time course of changes in refractive state and depth of vitreous chamber in response to the imposed myopic and hyperopic defocus. See Ref. [25] for details.

### RNA extraction and RNA-seq

After lens treatments, animals were humanely killed following IACUC-approved protocols. Both lens-treated and control eyes were enucleated, the retinae were dissected from the enucleated eyes, and the choroid/RPE was removed. The retinae were washed in RNAlater (Thermo Fisher Scientific, Grand Island, NY) for 5 minutes, frozen in liquid nitrogen, and stored at −80°C until processed for this study. To isolate RNA, tissue samples were homogenized at 4°C in a lysis buffer using Bead Ruptor 24 tissue homogenizer (Omni, Kennesaw, GA). Total RNA was extracted from each tissue sample using miRNAeasy mini kit (QIAGEN, Germantown, MD) following the manufacturer’s protocol. The integrity of RNA was confirmed by analyzing 260/280 nm ratios (Ratio_260/280_ = 2.11–2.13) on a Nanodrop (Thermo Fisher Scientific, Grand Island, NY) and the RNA Integrity Number (RIN = 9.0–10.0) using Agilent Bioanalyzer (Agilent, Santa Clara, CA). Illumina sequencing libraries were constructed from 1 μg of total RNA using the TruSeq Stranded Total RNA LT kit with the Ribo-Zero Gold ribosomal RNA depletion module (Illumina, San Diego, CA). The libraries, each containing a specific index (barcode), were pooled at equal concentrations using the randomized complete block (RCB) experimental design before sequencing on Illumina HiSeq 2500 sequencing system (Illumina, San Diego, CA). The number of libraries per multiplexed sample was adjusted to ensure sequencing depth of about 70 million reads per library (paired-end, 2 × 100 nucleotides). The actual sequencing depth was 70,851,538 ± 10,430,968 with read quality score 39.7 ± 0.2.

### Post-sequencing RNA-seq data validation and analysis

The FASTQ raw data files generated by the Illumina sequencing system were imported into Partek Flow software package (version 6.0.17.0723, Partek, St. Louis, MO), libraries were separated based on their barcodes, adapters were trimmed, and remaining sequences were subjected to pre-alignment quality control using Partek Flow pre-alignment QA/QC module. After the assessment of various quality metrics, bases with the quality score <37 were removed (≤5 bases) from each read. Sequencing reads were then mapped to the marmoset reference genome *C*. *jacchus* 3.2.1 (NCBI) using the STAR aligner (version 2.5.2b), resulting in 85.9% ± 2.4% mapped reads per library, which covered 36.9% ± 1.8% of the genome. Aligned reads were quantified to transcriptome using Partek E/M annotation model and the NCBI *C*. *jacchus* 3.2.1 annotation GFF file to determine read counts per gene/genomic region. The generated read counts were normalized by the total read count and subjected to the Partek Flow Gene Specific Analysis (GSA) to detect differentially expressed transcripts. Data were simultaneously fitted with Normal, Lognormal, Lognormal with shrinkage, Negative Binomial, or Poisson statistical models, and the best model was then applied to the corresponding subset of transcripts depending on the transcript expression within each subset. Differentially expressed transcripts were identified using a *P* value threshold of 0.05 adjusted for genome-wide statistical significance using Storey’s q-value algorithm [73]. Differential expression was calculated as fold change (FC, lens-treated eye versus control). To identify sets of genes with coordinate expression, differentially expressed transcripts were clustered using Partek Flow hierarchical clustering module using average linkage for the cluster distance metric and Euclidean distance metric to determine the distance between data points. Each RNA-seq sample corresponding to one control or one lens-treated eye was analyzed as a biological replicate, thus resulting in three biological replicates for control eyes and three biological replicates for lens-treated eyes in each of the four experimental groups.

### GO analysis and identification of sign-of-defocus–specific signaling pathways

To identify biological functions (GO categories), which are significantly affected by the changes in gene expression induced by the optical defocus in the retina, we used QIAGEN’s Ingenuity Pathway Analysis (IPA) software and database (QIAGEN, Germantown, MD). IPA Downstream Effects Analysis module was used to visualize biological trends and predict the effect of gene expression changes in the datasets on biological processes. Downstream Effects Analysis is based on expected causal effects between genes and functions; the expected causal effects are derived from the literature compiled in the Ingenuity Knowledge Base. The analysis examines genes in the dataset that are known to affect functions, compares the genes’ direction of change to expectations derived from the literature, and then issues a prediction for each function based on the direction of change. The direction of change was determined as the difference in gene expression in the retina exposed to +5D or −5D lenses relative to the contralateral control retina exposed to the plano lens. We used the Fisher's exact test with a *P* value threshold of 0.05 to estimate statistical significance and the z-score algorithm to make predictions about direction of change. The z-score algorithm is designed to reduce the chance that random data will generate significant predictions. The activation z‐score was used to infer likely activation states of biological functions based on comparison with a model that assigns random regulation directions. The z-score provided an estimate of statistical quantity of change for each biological function found to be statistically significantly affected by the changes in gene expression. The activation z‐score can also predict implicated biological functions independently from their associated *P* value, based on significant pattern match of up- or down-regulation. The activation z-score was also employed in the IPA Pathways Activity Analysis module to predict activation or suppression of the canonical pathways affected by optical defocus. The significance values for the canonical pathways were calculated by the right-tailed Fisher's exact test. The significance indicates the probability of association of molecules from each dataset with the canonical pathway by random chance alone. Pathways Activity Analysis determined if canonical pathways, including functional end points, are activated or suppressed based on differentially expressed genes or proteins in each dataset. Once statistically significant biological functions and canonical pathways for −5D/10 days, −5D/5 weeks, +5D/10 days, and +5D/5 weeks datasets were identified, we subjected these datasets to the Core Functional Analysis in IPA to compare the datasets and identify key similarities and differences in the biological functions and canonical pathways affected in each experimental group.

### Identification of candidate genes for human myopia

To identify candidate genes for human myopia, we compared the genes differentially expressed in the marmoset retina in response to imposed defocus with a list of genes located within QTLs found to be associated with myopia in a human population. We first compiled a list of all SNPs or markers exhibiting statistically significant association with myopia in the human linkage or GWAS studies. LDlink’s LDmatrix tool (National Cancer Institute) was used to identify SNPs in linkage disequilibrium and identify overlapping chromosomal loci. We then used UCSC Table Browser to extract all genes located within critical chromosomal regions identified by the human linkage studies or within 200 kb (±200 kb) of the SNPs found by GWAS. The list of genes located within human QTLs was compared with the list of genes that we found to be differentially expressed in the marmosets exposed to optical defocus using Partek Genomics Suite. The statistical significance of the overlaps was estimated using probabilities associated with the hypergeometric distribution, using Bioconductor software package GeneOverlap and associated functions.

## Supporting information

S1 Table List of genes differentially expressed in the retina of common marmosets exposed to −5D lens for 10 days. (XLSX)

S2 Table List of genes differentially expressed in the retina of common marmosets exposed to −5D lens for 5 weeks. (XLSX)

S3 Table List of genes differentially expressed in the retina of common marmosets exposed to +5D lens for 10 days. (XLSX)

S4 Table List of genes differentially expressed in the retina of common marmosets exposed to +5D lens for 5 weeks. (XLSX)

S5 Table Genetic networks associated with genes differentially expressed in the retina of common marmosets exposed to −5D lens for 10 days. (XLSX)

S6 Table Genetic networks associated with genes differentially expressed in the retina of common marmosets exposed to −5D lens for 5 weeks. (XLSX)

S7 Table Genetic networks associated with genes differentially expressed in the retina of common marmosets exposed to +5D lens for 10 days. (XLSX)

S8 Table Genetic networks associated with genes differentially expressed in the retina of common marmosets exposed to +5D lens for 5 weeks. (XLSX)

S9 Table Biological processes affected by the genes differentially expressed in the retina of common marmosets exposed to −5D lens for 10 days. (XLSX)

S10 Table Biological processes affected by the genes differentially expressed in the retina of common marmosets exposed to −5D lens for 5 weeks. (XLSX)

S11 Table Biological processes affected by the genes differentially expressed in the retina of common marmosets exposed to +5D lens for 10 days. (XLSX)

S12 Table Biological processes affected by the genes differentially expressed in the retina of common marmosets exposed to +5D lens for 5 weeks. (XLSX)

S13 Table Canonical pathways affected by the genes differentially expressed in the retina of common marmosets exposed to −5D lens for 10 days. (XLSX)

S14 Table Canonical pathways affected by the genes differentially expressed in the retina of common marmosets exposed to −5D lens for 5 weeks. (XLSX)

S15 Table Canonical pathways affected by the genes differentially expressed in the retina of common marmosets exposed to +5D lens for 10 days. (XLSX)

S16 Table Canonical pathways affected by the genes differentially expressed in the retina of common marmosets exposed to +5D lens for 5 weeks. (XLSX)

S17 Table Genes differentially expressed in the retina of common marmosets both after 10 days and after 5 weeks of exposure to +5D lens. (XLSX)

S18 Table Genes differentially expressed in the retina of common marmosets both after 10 days and after 5 weeks of exposure to −5D lens. (XLSX)

S19 Table Genes differentially expressed in the retina of common marmosets both after exposure to −5D and +5D lenses for 10 days. (XLSX)

S20 Table Genes differentially expressed in the retina of common marmosets both after exposure to −5D and +5D lenses for 5 weeks. (XLSX)

S21 Table List of myopia candidate genes located within critical chromosomal regions identified by human linkage studies or within 200 kb of the SNPs found by GWAS. GWAS, genome-wide association study.
(XLSX)

S22 Table Human chromosomal loci linked to myopia and candidate genes differentially expressed in marmosets exposed to +5D or −5D lenses. (XLSX)

S23 Table List of human myopia candidate genes differentially expressed in the retina of common marmosets exposed to +5D or −5D lenses for 10 days or 5 weeks. (XLSX)

S24 Table List of QTLs showing significant overlap with genes differentially expressed in the marmoset retina—odds ratios and *P* values. QTL, quantitative trait locus.
(XLSX)

S1 Fig Genetic network numbers 1–6 affected in the animals treated with −5D lenses for 10 days. Red, up-regulated in lens-treated eye. Green, down-regulated in lens-treated eye. See S5 Table for details.
(TIF)

S2 Fig Genetic network numbers 1–6 affected in the animals treated with −5D lenses for 5 weeks. Red, up-regulated in lens-treated eye. Green, down-regulated in lens-treated eye. See S6 Table for details.
(TIF)

S3 Fig Genetic network numbers 7–12 affected in the animals treated with −5D lenses for 5 weeks. Red, up-regulated in lens-treated eye. Green, down-regulated in lens-treated eye. See S6 Table for details.
(TIF)

S4 Fig Genetic network numbers 13–17 affected in the animals treated with −5D lenses for 5 weeks. Red, up-regulated in lens-treated eye. Green, down-regulated in lens-treated eye. See S6 Table for details.
(TIF)

S5 Fig Genetic network numbers 1–4 affected in the animals treated with +5D lenses for 10 days. Red, up-regulated in lens-treated eye. Green, down-regulated in lens-treated eye. See S7 Table for details.
(TIF)

S6 Fig Genetic network numbers 1–6 affected in the animals treated with +5D lenses for 5 weeks. Red, up-regulated in lens-treated eye. Green, down-regulated in lens-treated eye. See S8 Table for details.
(TIF)

S7 Fig Genetic network numbers 7–12 affected in the animals treated with +5D lenses for 5 weeks. Red, up-regulated in lens-treated eye. Green, down-regulated in lens-treated eye. See S8 Table for details.
(TIF)

S8 Fig Genetic network numbers 13–18 affected in the animals treated with +5D lenses for 5 weeks. Red, up-regulated in lens-treated eye. Green, down-regulated in lens-treated eye. See S8 Table for details.
(TIF)

S9 Fig Genetic network numbers 19–24 affected in the animals treated with +5D lenses for 5 weeks. Red, up-regulated in lens-treated eye. Green, down-regulated in lens-treated eye. See S8 Table for details.
(TIF)

S10 Fig Genetic network number 25 affected in the animals treated with +5D lenses for 5 weeks. Red, up-regulated in lens-treated eye. Green, down-regulated in lens-treated eye. See S8 Table for details.
(TIF)

## Abbreviations

AKT: Serine and Threonine kinase AKT
ARVO: Association for Research in Vision and Ophthalmology
BESOD: Bidirectional Emmetropization by the Sign of Optical Defocus
BRCA1: breast cancer 1 early onset
cAMP: cyclic adenosine monophosphate
CNTF: ciliary neurotrophic factor
CREB: cAMP responsive element binding protein
D: diopter
DARPP32: dopamine- and cAMP-regulated phosphoprotein 32 kDa
EIF2: eukaryotic translation initiation factor 2
eIF4: eukaryotic initiation factor 4
ERK: extracellular signal-regulated kinase
FC: fold change
GO: gene ontology
GSA: Gene Specific Analysis
GWAS: genome-wide association study
HIPPO: protein kinase Hippo
iNOS: inducible nitric oxide synthase
IPA: Ingenuity Pathway Analysis
JAK: Janus kinase
JNK: c-Jun N-terminal kinase
L: left
MAPK: mitogen-activated protein kinase
mTOR: mammalian target of rapamycin
NAD: nicotinamide adenine dinucleotide
NADH: reduced nicotinamide adenine dinucleotide
NGF: nerve growth factor; nNOS, neuronal nitric oxide synthase
PI3K: phosphatidylinositol 3-kinase
PTEN: phosphatase and tensin homolog
p70S6K: ribosomal protein S6 kinase
QTL: quantitative trait locus
R: right
RAN: Ras-related nuclear protein
RANK: receptor activator of nuclear factor kappa-B
RCB: randomized complete block
RIN: RNA Integrity Number
RNA-seq: massive parallel RNA sequencing
SAPK: stress-activated protein kinase
Stat: signal transducer and activator of transcription protein
TNFR1: tumor necrosis factor receptor 1
tRNA: transfer RNA
USDA: United States Department of Agriculture
Wnt: Wingless-integrated
6-OHDA: 6-hydroxydopamine
