# Supplementary figures and images for: Gene expression in response to optical defocus of opposite signs reveals bidirectional mechanism of visually guided eye growth
Source: PLoS Biol. 2018 Oct 9;16(10):e2006021. doi: 10.1371/journal.pbio.2006021 (PMC6177118; doi:10.1371/journal.pbio.2006021)

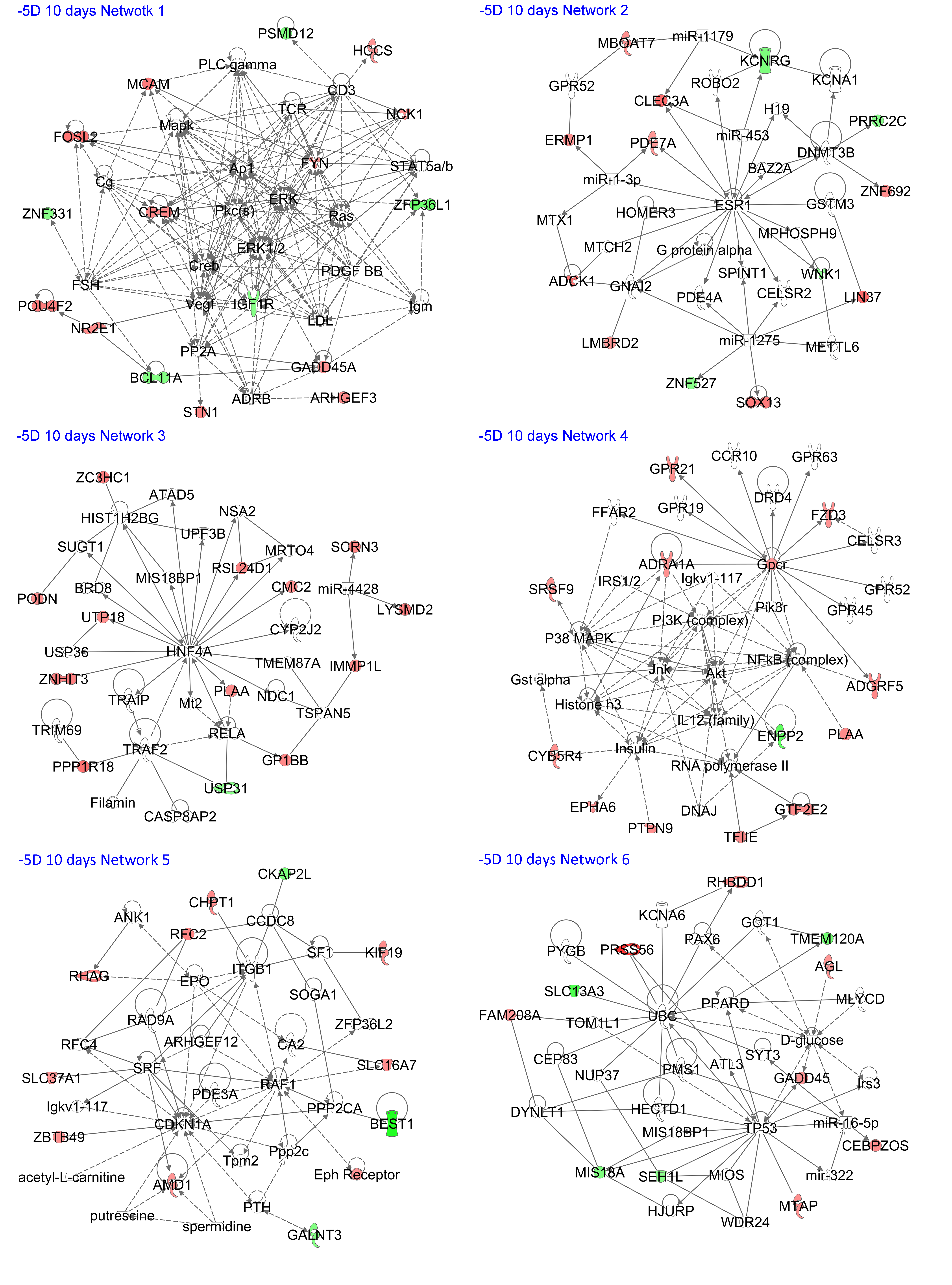

Supplement: S1 Fig — Red, up-regulated in lens-treated eye. Green, down-regulated in lens-treated eye. See S5 Table for details. (TIF) [file pbio.2006021.s025.tif]

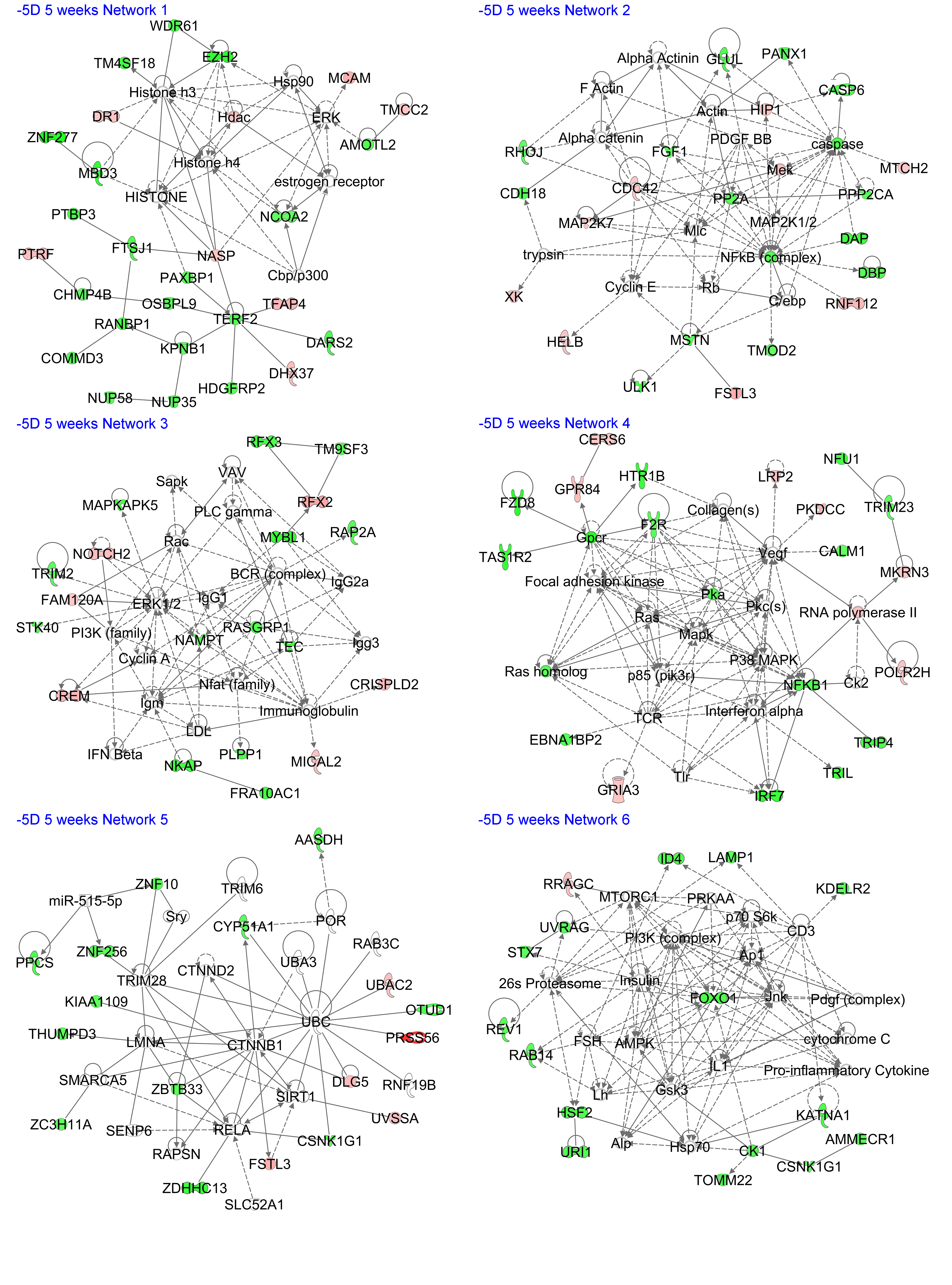

Supplement: S2 Fig — Red, up-regulated in lens-treated eye. Green, down-regulated in lens-treated eye. See S6 Table for details. (TIF) [file pbio.2006021.s026.tif]

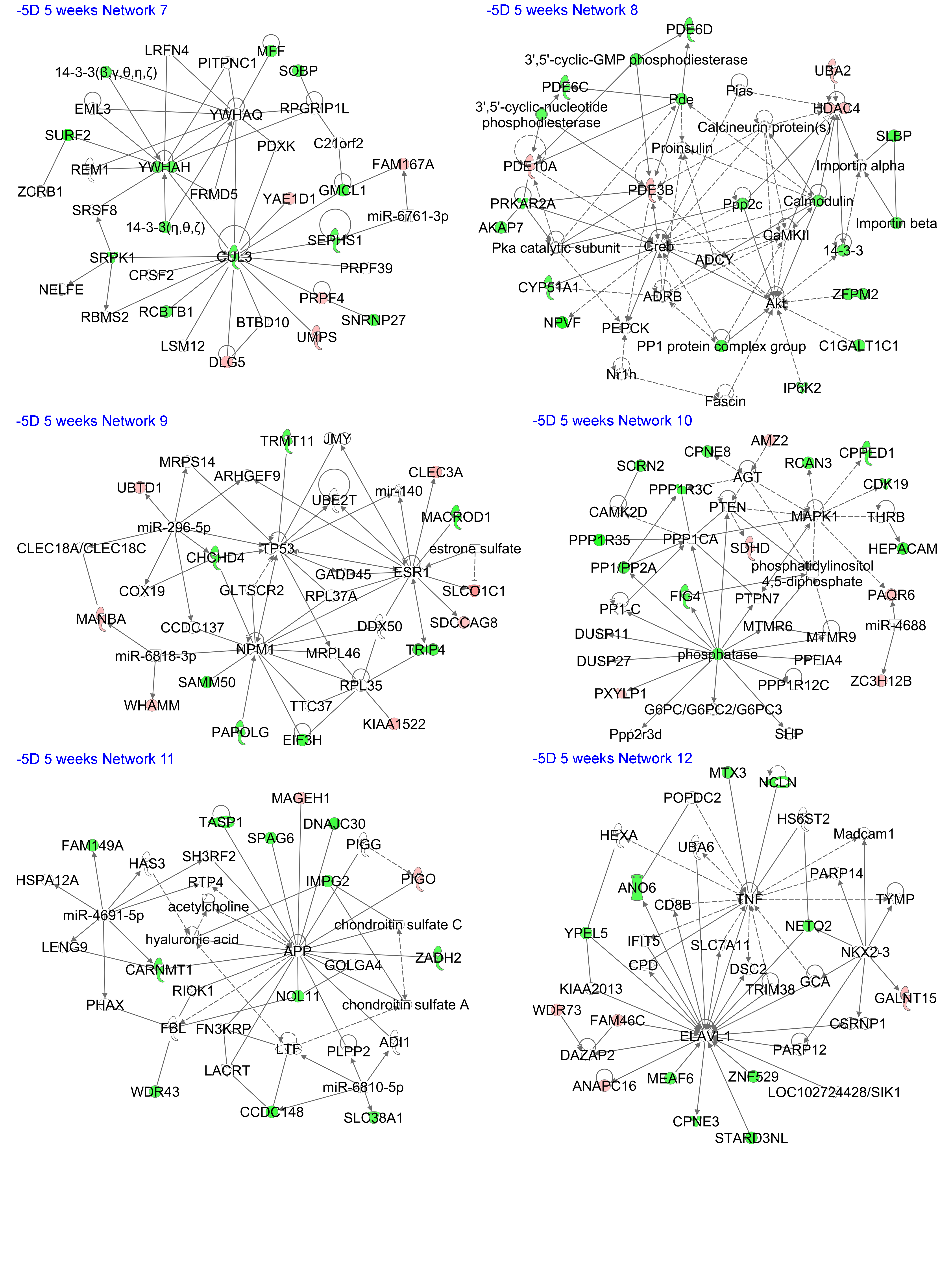

Supplement: S3 Fig — Red, up-regulated in lens-treated eye. Green, down-regulated in lens-treated eye. See S6 Table for details. (TIF) [file pbio.2006021.s027.tif]

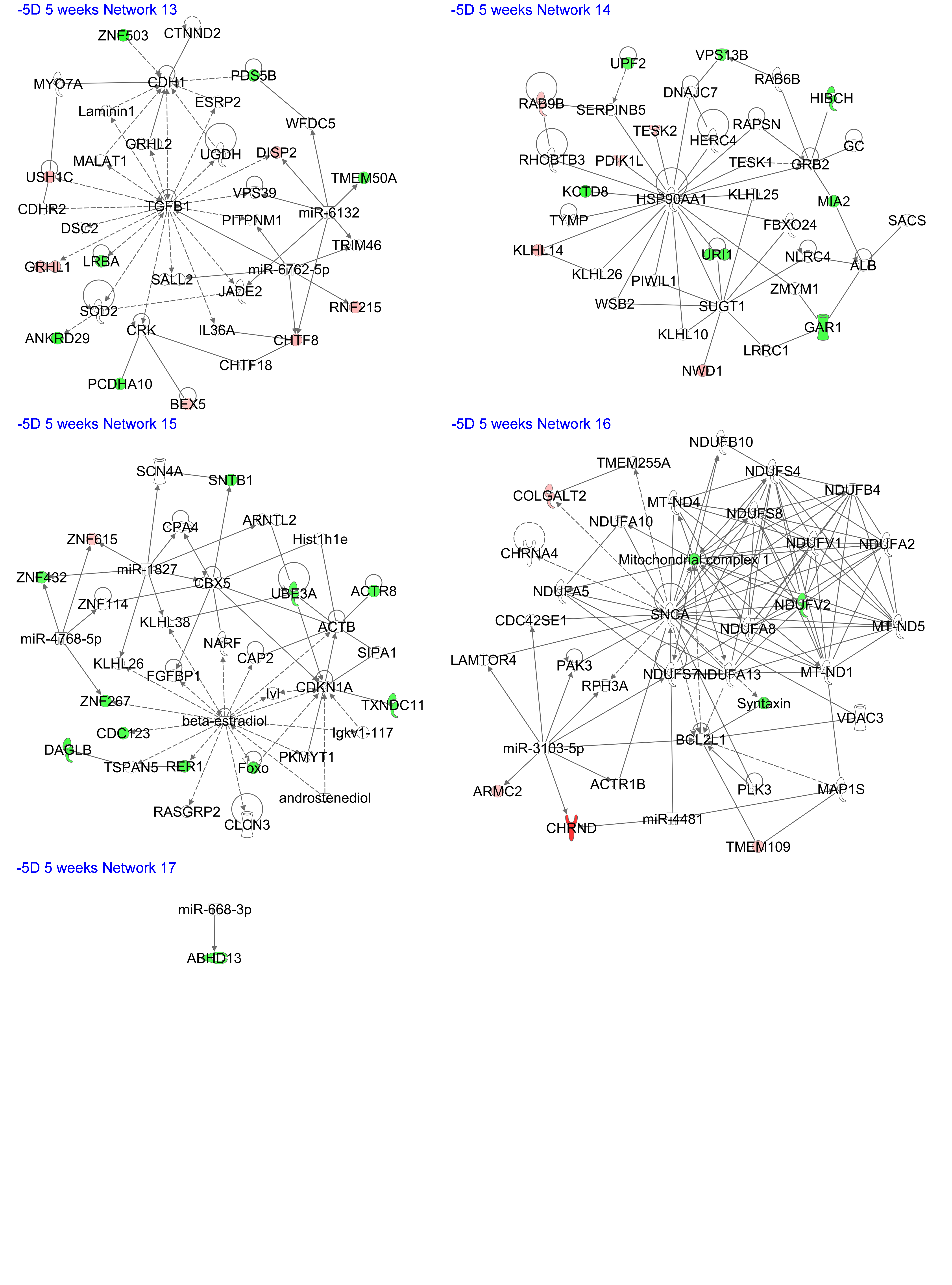

Supplement: S4 Fig — Red, up-regulated in lens-treated eye. Green, down-regulated in lens-treated eye. See S6 Table for details. (TIF) [file pbio.2006021.s028.tif]

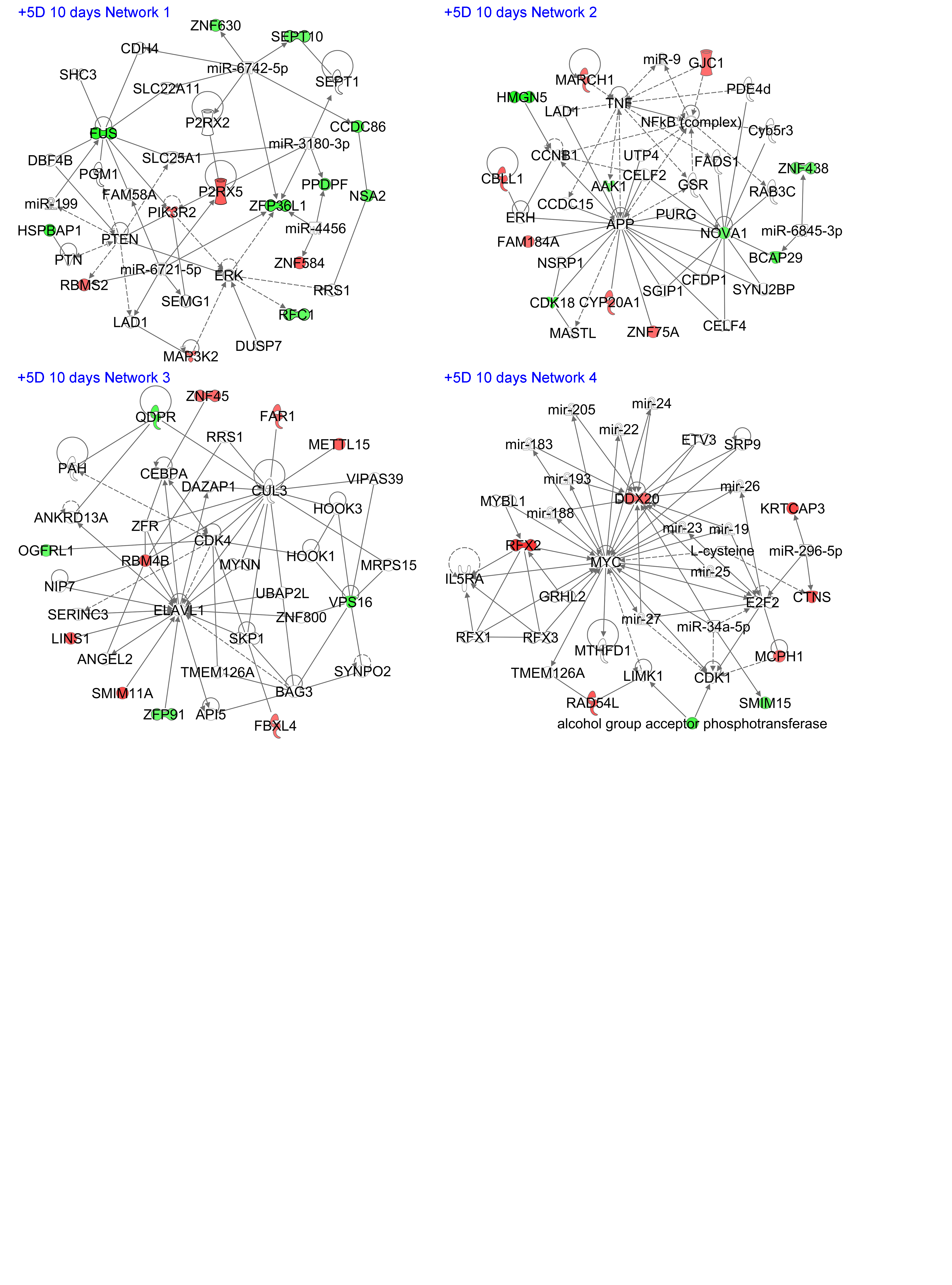

Supplement: S5 Fig — Red, up-regulated in lens-treated eye. Green, down-regulated in lens-treated eye. See S7 Table for details. (TIF) [file pbio.2006021.s029.tif]

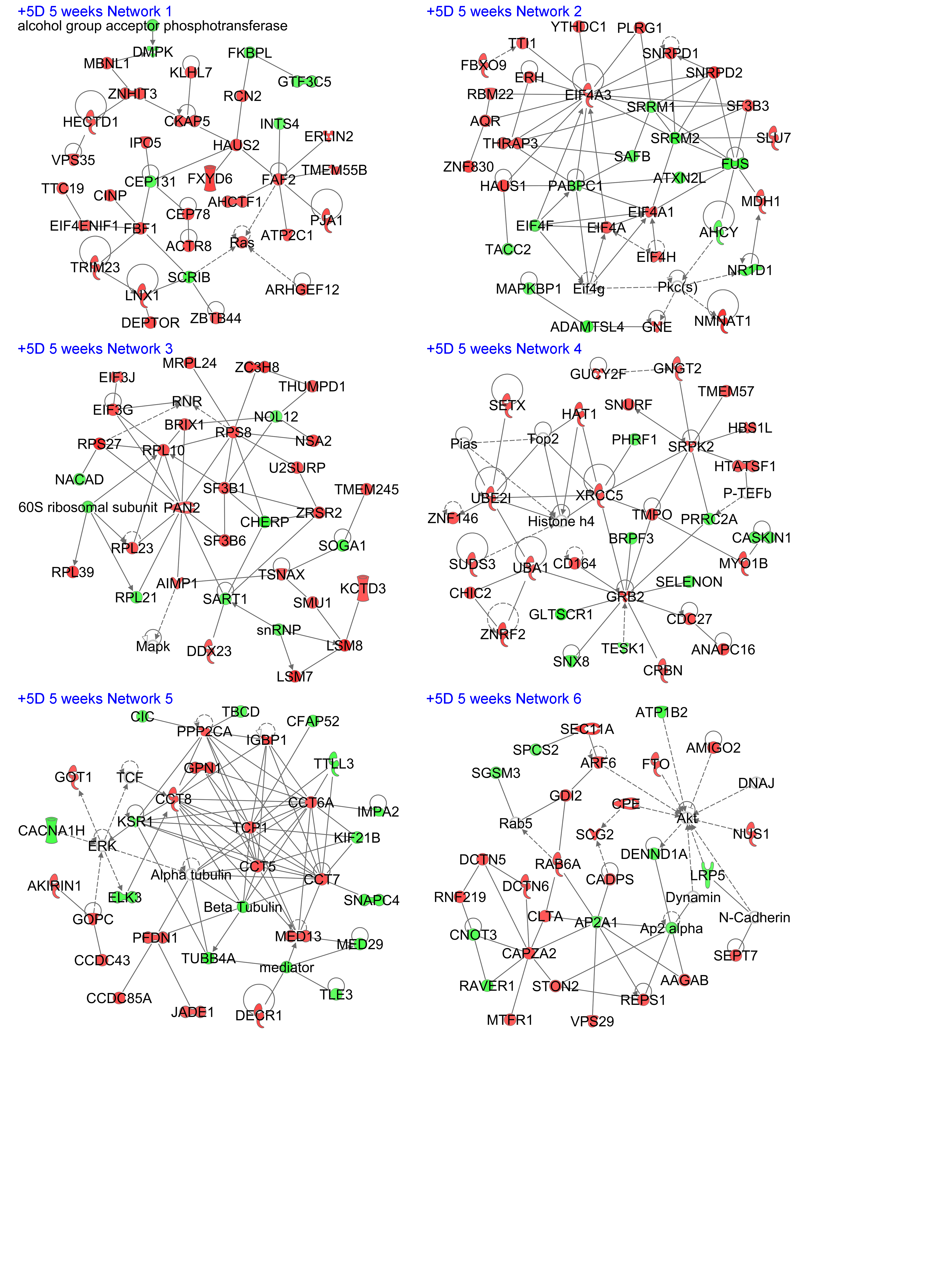

Supplement: S6 Fig — Red, up-regulated in lens-treated eye. Green, down-regulated in lens-treated eye. See S8 Table for details. (TIF) [file pbio.2006021.s030.tif]

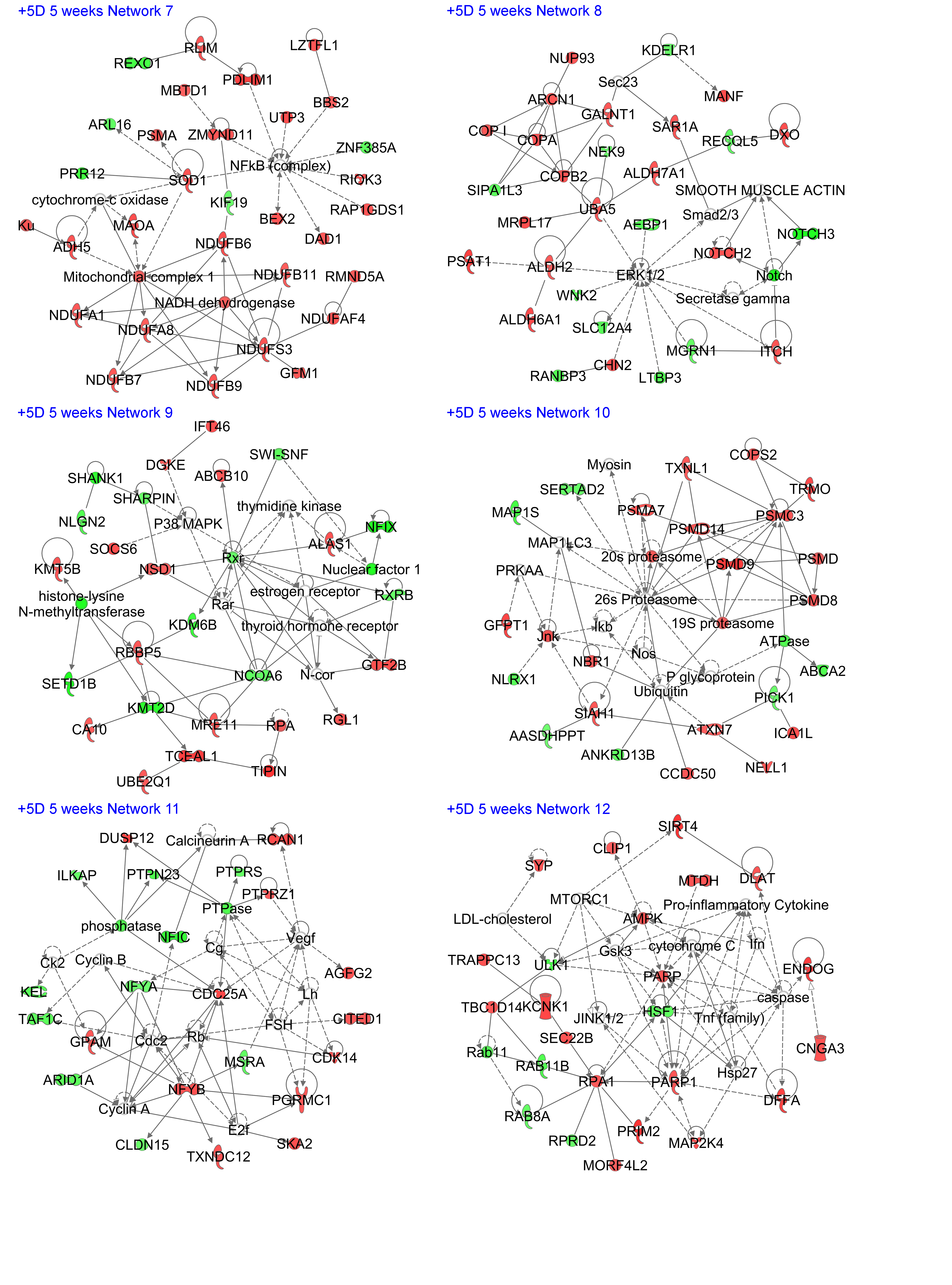

Supplement: S7 Fig — Red, up-regulated in lens-treated eye. Green, down-regulated in lens-treated eye. See S8 Table for details. (TIF) [file pbio.2006021.s031.tif]

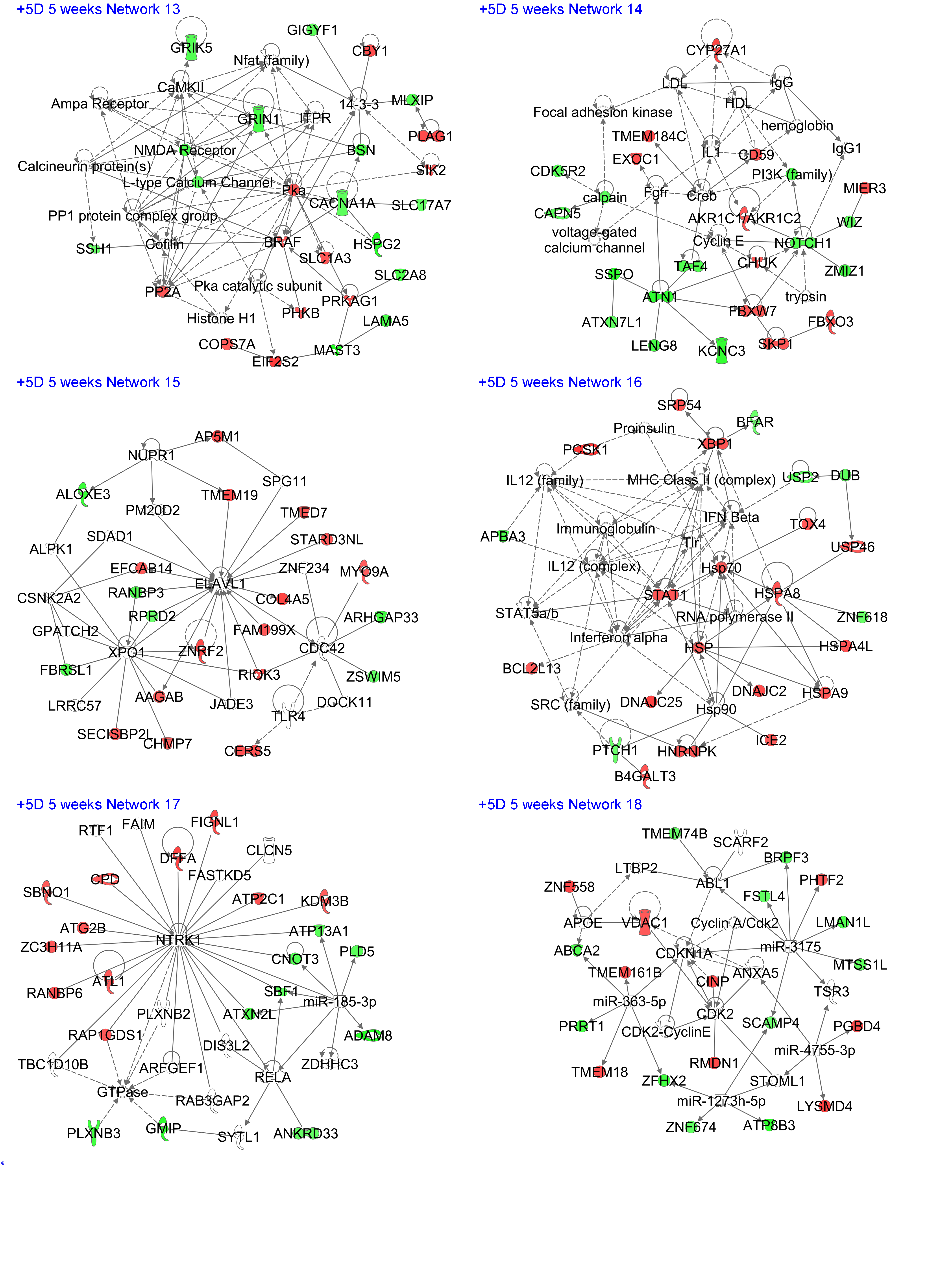

Supplement: S8 Fig — Red, up-regulated in lens-treated eye. Green, down-regulated in lens-treated eye. See S8 Table for details. (TIF) [file pbio.2006021.s032.tif]

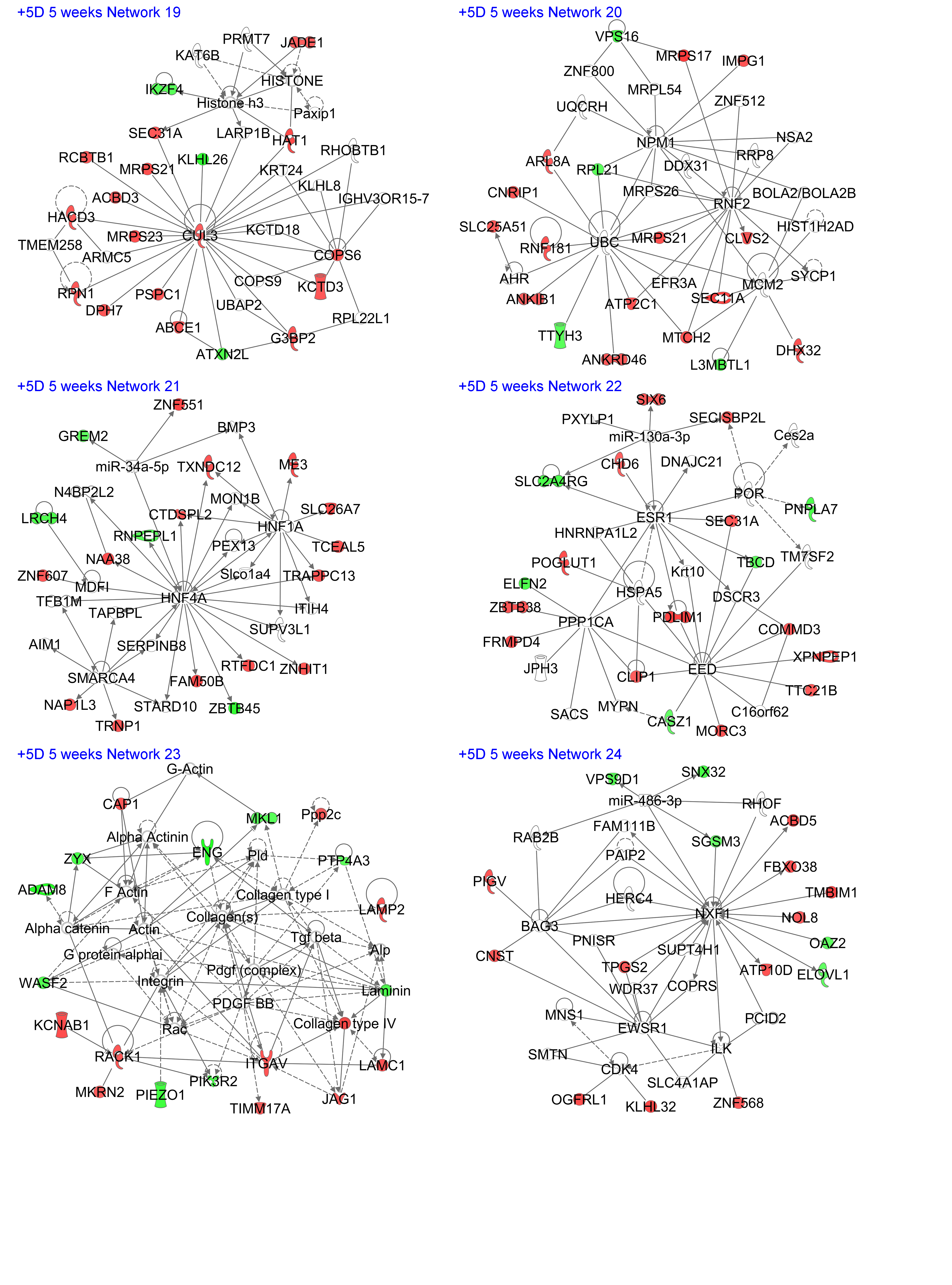

Supplement: S9 Fig — Red, up-regulated in lens-treated eye. Green, down-regulated in lens-treated eye. See S8 Table for details. (TIF) [file pbio.2006021.s033.tif]

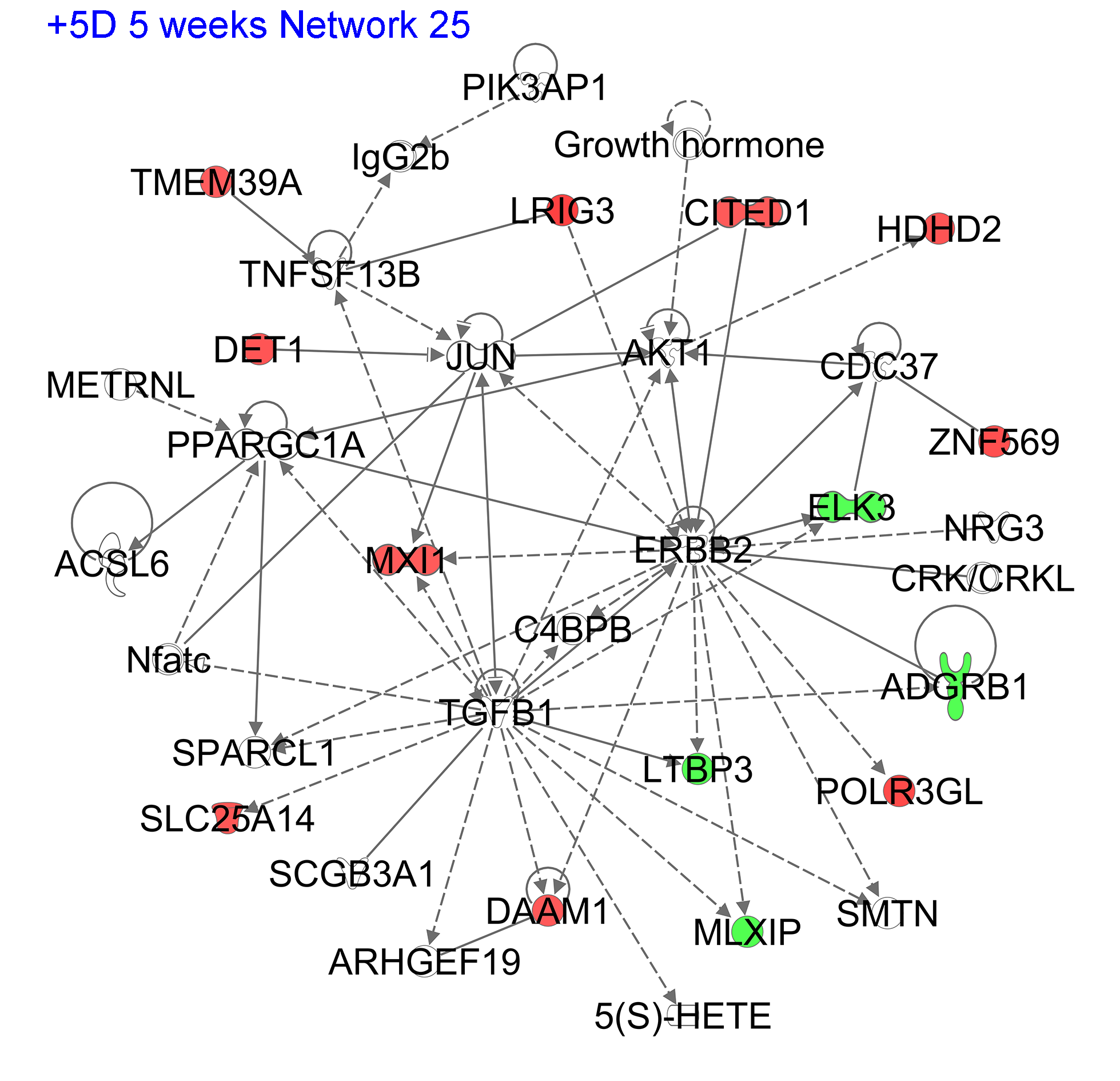

Supplement: S10 Fig — Red, up-regulated in lens-treated eye. Green, down-regulated in lens-treated eye. See S8 Table for details. (TIF) [file pbio.2006021.s034.tif]
